# Challenges and Opportunities for Rechargeable Aqueous Sn Metal Batteries

**DOI:** 10.1002/adma.202417757

**Published:** 2025-03-13

**Authors:** Haozhe Zhang, Di‐Jia Liu, Kang Xu, Ying Shirley Meng

**Affiliations:** ^1^ Pritzker School of Molecular Engineering The University of Chicago 5801 South Ellis Avenue Chicago IL 60637 USA; ^2^ Chemical Sciences and Engineering Division Argonne National Laboratory 9700 S Cass Avenue Lemont IL 60439 USA; ^3^ SES AI Corporation 35 Cabot Road Woburn MA 01801 USA; ^4^ Energy Storage Research Alliance Argonne National Laboratory 9700 South Cass Avenue Lemont IL 60439 USA

**Keywords:** anode, aqueous batteries, electrodeposition, reversibility, Sn metal

## Abstract

Rechargeable aqueous batteries based on metallic anodes hold tremendous potential of high energy density enabled by the combination of relatively low working potential and large capacity while retaining the intrinsic safety nature and economical value of aqueous systems; However, the realization of these promised advantages relies on the identification of an ideal metal anode chemistry with all these merits. In this review, the emerging Sn metal anode chemistry is examined as such an anode candidate in both acidic and alkaline media, where the inertness of Sn toward hydrogen evolution, flat low voltage profile, and low polarization make it a unique metal anode for aqueous batteries. From a panoramic viewpoint, the key challenges and detrimental issues of Sn metal batteries are discussed, including dead Sn formation, self‐discharge, and electrolyte degradation, as well as strategies for mitigating these issues by constructing robust Sn anodes. New design approaches for more durable and reliable Sn metal batteries are also discussed, with the aim of fully realizing the potential of Sn anode chemistry.

## Graphical abstract

Rechargeable aqueous batteries with metal anodes promise enhanced energy density, combining higher output voltage and capacity with high safety. This perspective highlights the emerging potential of the Sn metal anode, emphasizing its resistance to hydrogen evolution, high reversibility, and sustainability. Key challenges, including dead Sn formation and electrolyte evolution, as well as innovative design strategies for robust Sn‐based batteries are also discussed. The development of Sn metal batteries could contribute to a sustainable society.

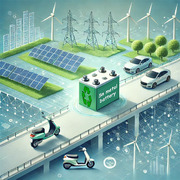

## 1 Introduction

The growing demand for sustainable energy sources has created an urgent need for reliable and safe energy storage systems.^[^ ^1^ ^]^ While lithium‐ion battery dominates today's energy storage technologies in powering literally everything from small appliances to electric vehicles and grid, it faces limitations in operation safety, resource scarcity, and high costs, especially for tera‐watt‐hour energy storage demands projected in the near future.^[^ ^2^ ^]^ Rechargeable aqueous batteries (RABs), which utilize water‐based electrolytes with intrinsic incombustibility and high specific heat capacity, have attracted increasing attention by promising inherent benefits such as high safety, low cost, manufacturing simplicity and convenience, and environmentally friendliness.^[^ ^3^ ^]^ These features make RABs ideal for stationary grid‐level energy storage, which could tolerate somewhat lower gravimetric and volumetric energy densities when compared to portable and electric mobility applications.^[^ ^4^ ^]^ Up to now, RABs occupy approximately half of the rechargeable battery market. However, the mediocre energy density (<50 Wh kg⁻^1^) and poor cycle life (<500 cycles) of these batteries developed decades ago (e.g., Pb‐acid, Ni‐MH)^[^ ^5^ ^]^ become increasingly unable to satisfy the automotive and grid energy storage application needs.^[^ ^6^ ^]^

Enhancing the energy density and output voltage of RABs remains the most formidable challenge, which is confined by the electrochemical stability windows of aqueous electrolytes (1.23 V) as defined by the potentials of oxygen‐ and hydrogen‐evolution reactions (OER and HER), respectively.^[^ ^7^ ^]^ Metal anode materials constitute the key to achieving these goals because they provide low and flat electrode potential, high specific capacity, and most importantly, potentially high overpotential for HER, suppressing hydrogen formation on anode surface below 0 V versus RHE to expand RAB's operation voltage.^[^ ^8^ ^]^ Among various metals, those based on plating/stripping electrochemistry (e.g., Zn,^[^ ^9^ ^]^ Cu,^[^ ^10^ ^]^ Fe,^[^ ^11^ ^]^ Sb,^[^ ^12^ ^]^ Mn,^[^ ^13^ ^]^ Al,^[^ ^14^ ^]^ Cd^[^ ^15^ ^]^) have emerged as promising candidates because of faster reaction kinetics. To date, a series of metal batteries based on Zn anode have become the focus of attention.^[^ ^16^ ^]^ However, most of the anodes in an aqueous system (especially in extreme pH conditions) still suffer from some fatal flaws such as dendrite formation, self‐corrosion, passivation, and especially hydrogen formation, which lead to poor Coulombic efficiency, limited cycling life and safety issue.^[^ ^17^ ^]^

As an alternative to Zn, Tin (Sn) recently emerged as a competitive RAB anode due to its relatively low cost, nontoxicity, good recyclability, and reasonably high specific capacity by its bivalence/tetravalence of ionic forms.^[^ ^18^ ^]^ The most important advantage of Sn lies in its high acid tolerance, which renders its operational stability in both acidic and alkaline environments, where other metal anodes, such as Zn and Fe, experience sustained corrosion and degradation.^[^ ^19^ ^]^ Unlike Zn, metallic Sn possesses a body‐centered tetragonal crystal structure with isotropic surface energy but high surface anisotropy, which promotes preferential electrodeposition as polyhedron particles hence less susceptible to dendrite formation.^[^ ^20^ ^]^ With aqueous tin metal batteries (SnMBs) still in their infancy, several challenges remain unresolved. A key issue is a formation of “dead Sn”, whose accumulation directly leads to loss of active materials and subsequent capacity fade over repeated cycling. Meanwhile, the competitive HER on the Sn surface and the accompanied electrolyte consumption severely reduce the practicality of the Sn metal anode. Although certain improvements have been achieved via electrolyte and interfacial engineering in attempts to fully unlock tin's potential, its performance is still far from being satisfactory.

In this perspective, we aim at capturing the recent efforts made on Sn metal anode in aqueous electrolyte and envision the path forward. We first review the fundamental electrochemistry underneath Sn anode material, its development history, unique characteristics, and recent advancements, with emphasis placed on a comprehensive summary of its material innovations and battery system designs. We then explore future opportunities for such chemistry in both acidic and alkaline systems and offer our projection on its future directions and potential role in the wide energy storage landscape.

## 2 Fundamentals of Sn Metal Batteries

**Figure 1:**
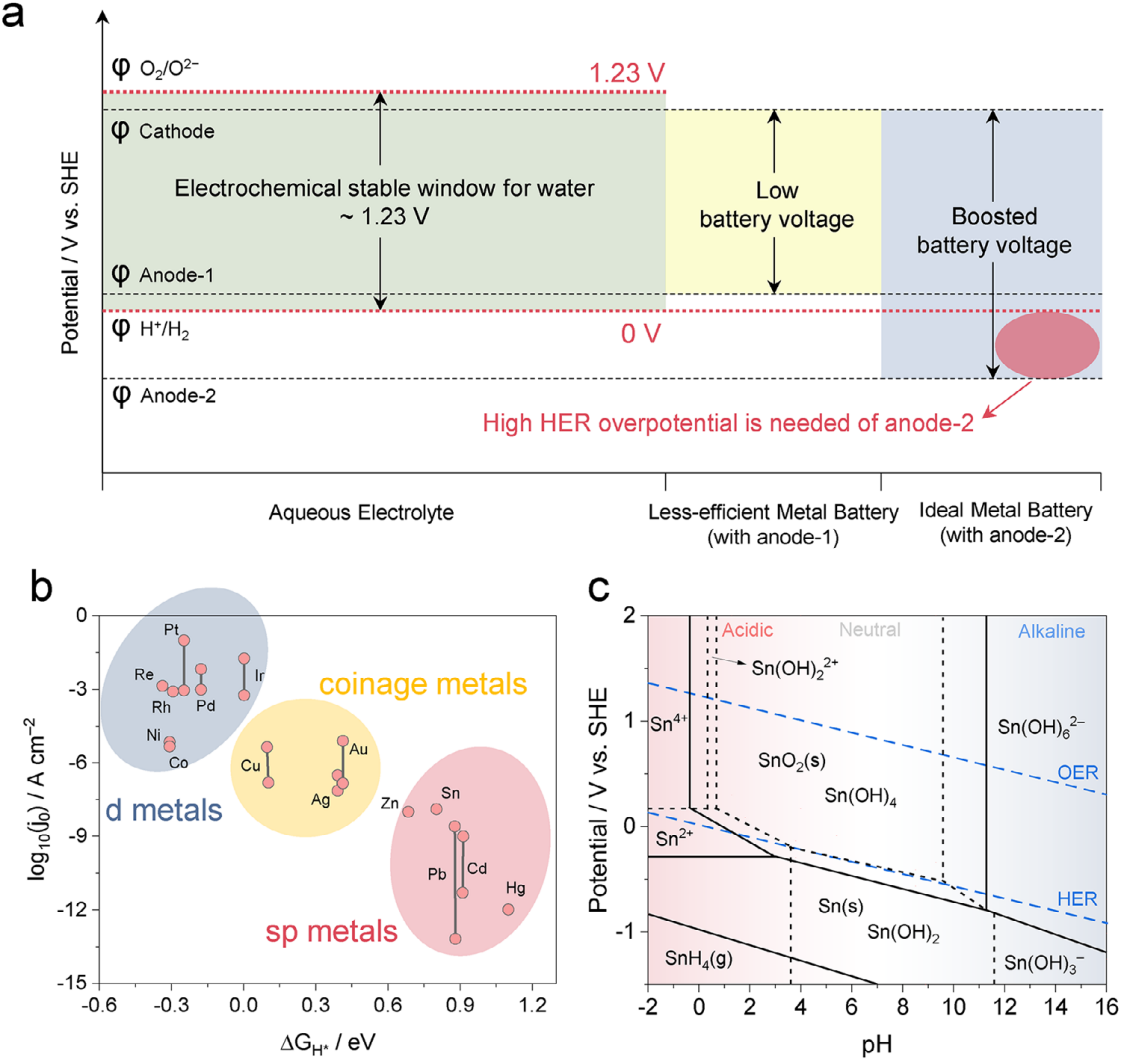
a) The electrochemical stability window of aqueous electrolytes. The overpotential against HER of anode could expand the electrochemical operating window. b) The comparison of HER on various metal surfaces in acidic aqueous solutions. The trends in acid and alkaline media are similar. Experimental HER activity is expressed as the exchange current density, log(j_0_), as a function of the calculated H^*^ Gibbs energy, ΔG_H*_. c) Pourbaix diagram of the H_2_O–Sn system at 298 K. Dissolved Sn activity is 10^−6^ and p(SnH_4_) = 1. The solid line means where solid exists in equilibrium with the adjacent species, and the dashed line means available Sn species in solution are equally distributed in the two adjacent zones.

Since the very first demonstration in the 1860s, RABs have been the workhorse that powered the industrial revolution.^[^ ^21^ ^]^ Even in the era after Li‐ion batteries, RABs maintained their footprint in extensive applications from household to electric mobility and electric grids. The combined merits of their relatively low manufacturing requirements, earth‐abundant and cheap raw materials, low maintenance, and minimized system packaging render them the most cost‐effective system.^[^ ^2b^ ^]^ However, their major limit are also apparent: low output voltage (mostly < 1.5 V) and consequently low output energy.^[^ ^22^ ^]^ Pure water under thermodynamic equilibrium has a narrow electrochemical stability window of 1.23 V, dictated by HER and OER redox reactions. (green area, **Figure** **1**a). This feature by thermodynamics constrains the operation window of the RABs (yellow area). However, if a certain reaction on an electrode experiences a kinetical barrier, the actual onset potential could be pushed away from the thermodynamic equilibrium, resulting in a phenomenon known as “polarization” in electrochemistry, which in fact expands the operational voltage window. In reality, the overpotentials of HER and OER, as well as the electrolytes of different compositions will expand the actual operation window. Between these two reactions associated with the decomposition of water, OER often suffers a higher kinetic barrier given its four‐electron nature, while HER typically encounters little polarization. Hence for a metal anode to operate beyond the voltage limits allowed by its thermodynamics, one expects anode materials to have high overpotentials for HER, so that the undesirable hydrogen production can be suppressed, and the working voltage of the RABs can be effectively expanded (blue area). Generally speaking, anode candidates that meet such expectations are metals of low redox potential (<0 V vs RHE) and high specific capacity. When the redox potential of M^n+^/M resides below HER, the hydrogen evolution becomes competing reactions with metal deposition, which inevitably causes low coulombic efficiency and severe hydrogen gas formation. Although aqueous electrolytes are intrinsically non‐flammable, continuous water decomposition and hydrogen accumulation could lead to an alternative safety hazard.^[^ ^23^ ^]^

Hence high intrinsic HER overpotential on metal anode is essential in preventing hydrogen formation, which not only ensures higher Coulombic efficiency at more negative potentials allowed by Pourbaix limits but also mitigates the self‐discharge.^[^ ^24^ ^]^ An HER at a metal surface typically involves two key steps: the electrochemical proton or hydronium adsorption and subsequent charge‐transfer (Volmer step) followed by either a sequential electrochemical proton‐electron transfer desorption (Heyrovsky step) or a synergistic chemical desorption of H_2_ by combing two adsorbed H atoms (Tafel step), as listed below:^[^ ^25^ ^]^

(1) Electrochemical adsorption (Volmer step)

(1) $$H_{3}O^{+}+M+e^{-}↔M-H^{*}+H_{2}O\;(\mathrm{in}\;\mathrm{acid})$$

(2) $$H_{2}O+M+e^{-}↔M-H^{*}+OH^{-}\;\left(\mathrm{in}\;\mathrm{alkaline}\right)$$

(2) Electrochemical desorption (Heyrovsky step)

(3) $$H^{+}+M-H^{*}+e^{-}↔M+H_{2}\;\left(\mathrm{in}\;\mathrm{acid}\right)$$

(4) $$H_{2}O+M-H^{*}+e^{-}↔M+OH^{-}+H_{2}\;\left(\mathrm{in}\;\mathrm{alkaline}\right)$$

Or

(3) Chemical desorption (Tafel step)

(5) $$2M-H^{*}↔2M+H_{2}\;(\mathrm{in}\;\mathrm{both}\;\mathrm{acid}\;\mathrm{and}\;\mathrm{alkaline})$$

In addition, the HER process in neutral and near‐neutral electrolytes with low H_3_O⁺ concentration will proceed with a hybrid reduction mechanism due to the involvement of both H_2_O molecules and dissociated H_3_O⁺ ions. In neutral conditions, H_3_O⁺ ions will serve as the primary reactants at low overpotentials near the equilibrium potential (0 V vs RHE). The reaction rate is slow due to the limited H_3_O⁺ concentration to sustain the process. As overpotentials increase, rapid H_3_O⁺ consumption near the electrode surface creates a localized pH gradient, leading to diffusion‐controlled kinetics. The direct reduction of H_2_O molecules is thermodynamically unfavorable at low overpotentials, as it requires a higher overpotential than H_3_O⁺ reduction. At higher overpotentials, the dominant reactants will shift from H_3_O⁺ ions to H_2_O molecules. Consequently, the HER activity of metals in acidic and alkaline conditions is much higher than that in neutral conditions, so the development of metal anodes in extreme pH conditions is more challenging.

The HER overpotential depends heavily on the efficiency of the above reactions.^[^ ^26^ ^]^ The Sabatier principle states that for a catalyst to be effective, the interaction between the catalyst and the reactive intermediates must be optimized.^[^ ^27^ ^]^ Both the effectiveness of hydrogen adsorption (H^*^) and hydrogen desorption (H_2_) can be quantified by the Gibbs free energy of hydrogen adsorption (ΔG_H*_).^[^ ^28^ ^]^ For H_2_ generation, a near‐optimal ΔG_H*_ ensures that hydrogen is adsorbed just strongly enough to promote the reaction but can also desorb readily. Therefore, the ΔG_H*_ value of the metal anode candidate must be deviated from zero to prohibit the HER.^[^ ^29^ ^]^ If this interaction on the metal anode surface is too weak (ΔG_H*_>0), insufficient intermediates will adsorb onto the surface, leading to sluggish HER kinetics and high overpotential. On the other hand, if the interaction is too strong (ΔG_H*_<0), the reaction products will remain bound to the surface, preventing further reaction by occupying the active sites and also resulting in high HER overpotential.^[^ ^30^ ^]^

In general, the HER inertness of metal anode can be predicted by its intrinsic electronic band structures. The relationships between the ΔG_H*_ values and the HER performance represented by the exchange current density j_0_ over common metals are summarized in Figure 1b.^[^ ^31^ ^]^ It shows that the d‐metals are not suitable for metal anodes due to their fast HER kinetics. The Fermi level of the d‐metals lies within the d‐band, which is crucial for bonding H^*^ onto the metal surface during the Volmer step of the HER with negative ΔG_H*_ values.^[^ ^32^ ^]^ Moderate d‐orbital occupancy will render some d‐metal such as Pt and Pd with balanced adsorption and desorption energy of hydrogen, resulting in unsatisfactory anode suitability due to high HER activity.^[^ ^33^ ^]^ In coinage metals like Cu, Ag, and Au, the d‐band center gradually shifts away from the Fermi level resulting in relatively weak binding to hydrogen, but their HER inertness is still not sufficient for an anode candidate. The sp metals are selected to expand the HER overpotential due to their electronic structure. The ΔG_H*_ of sp metal is extremely high because their d band lies so low that it plays no role in the bonding of hydrogen, and the sp band at the Fermi level does not overlap with H 1s orbital effectively. As a result, metals like Pb, Hg, Sn, and Zn have high HER overpotential and could be used as anode candidate for aqueous batteries.^[^ ^34^ ^]^

Sn metal is a typical sp metal with a long history of serving civilization since 3000 BC.^[^ ^35^ ^]^ Pure Sn has a silver‐grey metallic luster and good tensile properties and has been widely‐used as solder, alloy component, anti‐corrosion protect layer, etc.^[^ ^36^ ^]^ It has a relatively low price (ca. 23 $ kg^−1^)^[^ ^37^ ^]^ and a stable global supply chain,^[^ ^38^ ^]^ because it is relatively abundant in the earth's crust (2.3 ppm) and is distributed among every continent.^[^ ^39^ ^]^ The recycling process of Sn is very mature, with more than 35% of tin products being recycled.^[^ ^40^ ^]^ In addition, Sn is nontoxic, unlike most of its sp metal counterpart such as Pb, Cd, and Hg.^[^ ^41^ ^]^ The manufacturing process of Sn metal from Cassiterite (SnO_2_) also has a lower environmental impact compared to other metals widely used in batteries such as cobalt. These sustainable features of Sn make it a viable candidate for large‐scale energy storage applications where the environmental impact is an important consideration.

The ideal metallic anode needs to meet the essential kinetics, capacity, and redox potential requirements for RAB performance.^[^ ^8^, ^17^ ^]^ The metal anode used in aqueous batteries can be divided into two types based on the electron storage mechanism.^[^ ^12^ ^]^ The first type is “solid conversion metal” such as Pb,^[^ ^42^ ^]^ Bi,^[^ ^43^ ^]^ and Cd.^[^ ^44^ ^]^ These metals undergo phase conversion from metal to insoluble compounds, such as metal hydroxides and sulfate, during the energy storage process. Poor conductivity and slow reaction kinetics caused by these compounds lead to low capacity and limited rate capability.^[^ ^45^ ^]^ In contrast, the second type plating/stripping metals such as Zn and Sb, avoid these issues by relying on the reversible transformation between metal and metal ions during the charge/discharge cycle, thus achieving fast reaction kinetics.^[^ ^46^ ^]^ The H_2_O–Sn Pourbaix diagram at 25 °C in Figure 1c shows that Sn exists in metallic form under all pH or soluble ionic form under extreme pH (<4 or >9), suggesting that Sn could serve as plating/stripping‐type anode in both acidic (Sn/Sn^2+^ or Sn/Sn^4+^) and alkaline (Sn/Sn(OH)_3_⁻, trihydroxostannite ion or Sn/Sn(OH)_6_^2^⁻, hexahydroxostannate ion) batteries.^[^ ^47^ ^]^

**Figure 2:**
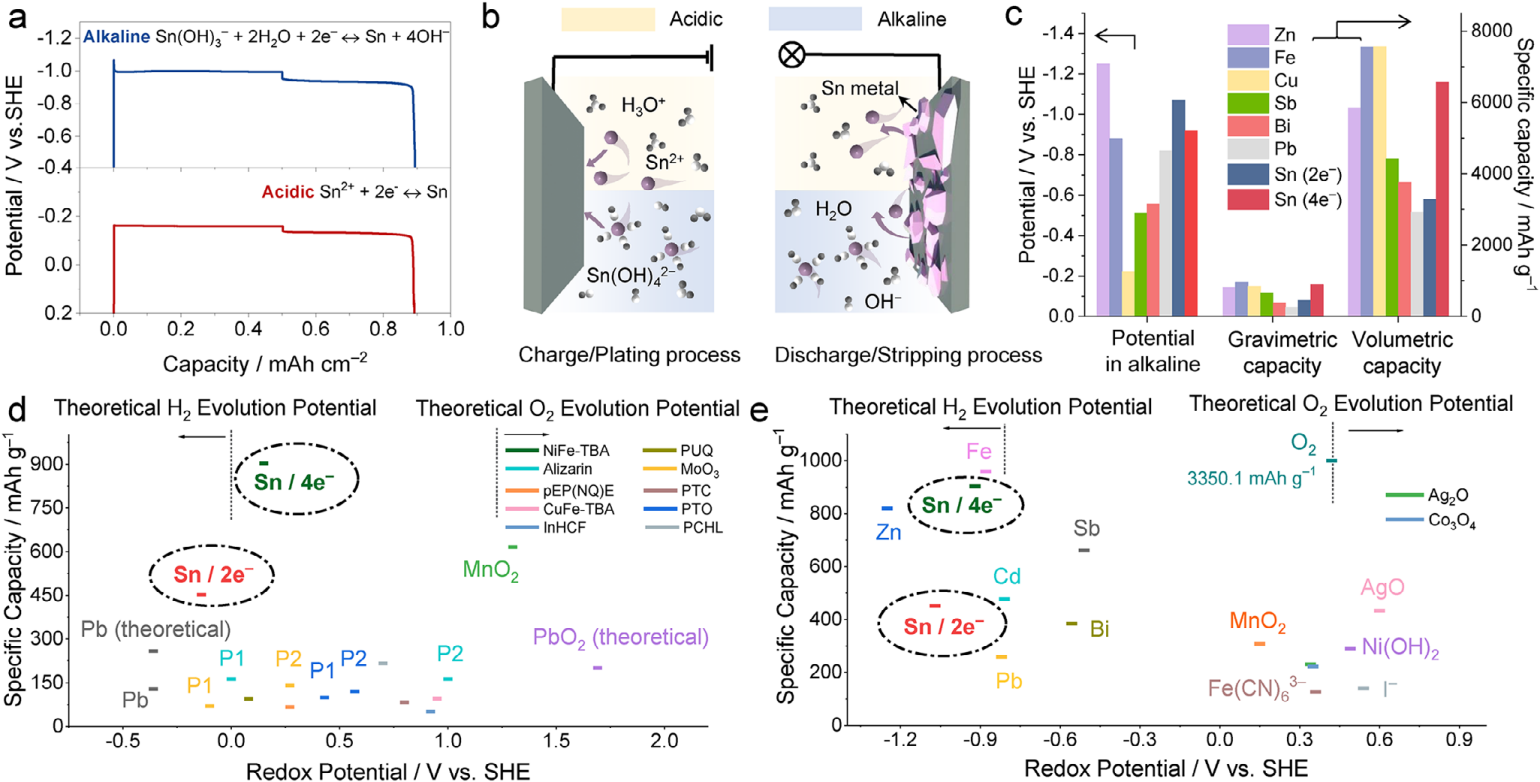
a) Experimental data of galvanostatic charge–discharge curves of Sn metal anode in typical acidic and alkaline electrolytes under the current density of 2 mA cm^−2^. b) Schematic illustration of the charge storage mechanism of the Sn anode in acidic and alkaline electrolyte. The anions are omitted. c) Redox potential and gravimetric and volumetric specific capacities of typical metal anode for aqueous battery. Redox potentials and specific capacities of Sn metal anode and typically reported electrodes in d) acidic^[^ ^3^, ^49^ ^]^ and e) alkaline system.^[^ ^20^, ^50^ ^]^ P1 and P2 indicate the first and the second plateau.

**Figure** **2**a illustrates galvanostatic charge–discharge experiments conducted based on two electron transfer Sn chemistry in both acidic (red line, 1 M H_2_SO_4_ + 0.2 M SnSO_4_) and alkaline (blue line, 3 M KOH + 0.2 M SnSO_4_) electrolytes with graphite paper as a current collector. The current density is 2 mA cm^−2^, and the deposition capacity is 0.5 mAh cm^−2^. Both Sn electrodes in acidic and alkaline systems show stable, flat voltage profiles during the plating/striping process with low polarization, validating Sn metal as an anode for both aqueous acidic and alkaline batteries (Figure 2b).

The multiple‐electron transfer capability and its high density (7.31 g cm^−3^) enable SnMBs of a relatively high specific gravimetric (451.6 mAh g^−1^ based on 2e^−^ and 903.2 mAh g^−1^ based on 4e^−^) and volumetric (3281.3 mAh cm^−3^ based on 2e^−^ and 6562.5 mAh cm^−3^ based on 4e^−^) capacity.^[^ ^48^ ^]^ As shown in Figure 2c, Sn metal exhibits advantages over most aqueous metal anodes in terms of redox potential and specific capacity. The energy advantages are detailly shown when we list the profiles of commonly used cathode and anode materials in acidic (Figure 2d)^[^ ^3^, ^49^ ^]^ and alkaline (Figure 2e)^[^ ^20^, ^50^ ^]^ battery systems. The acidic battery represented by the lead‐acid battery is one of the most widely used batteries in the world. However, its performance is severely limited by the anode materials like lead. Many traditional battery anodes using metal/metal compounds could not survive in acid, which limits the material selection. Furthermore, most metal electrode potentials fall within the range of 0.2 to 1.0 V versus SHE, limiting their pairing ability to full‐cell configurations of high voltage output. To this end, the Sn metal electrode stands out with its negative potential and high capacity, allowing it to pair with a wide range of electrodes to achieve output voltages ranging from over 0.5 V up to 1.8 V. In the alkaline system, the application of tin metal has challenged zinc metal as the best anode candidate in aqueous battery. Metallic Sn shows compatible energy to that of Zn metal batteries along with the intrinsic HER inertness and dendrite‐less feature, which may achieve better cycling performance.

## 3 Roadmap of Sn Metal Batteries

**Figure 3:**
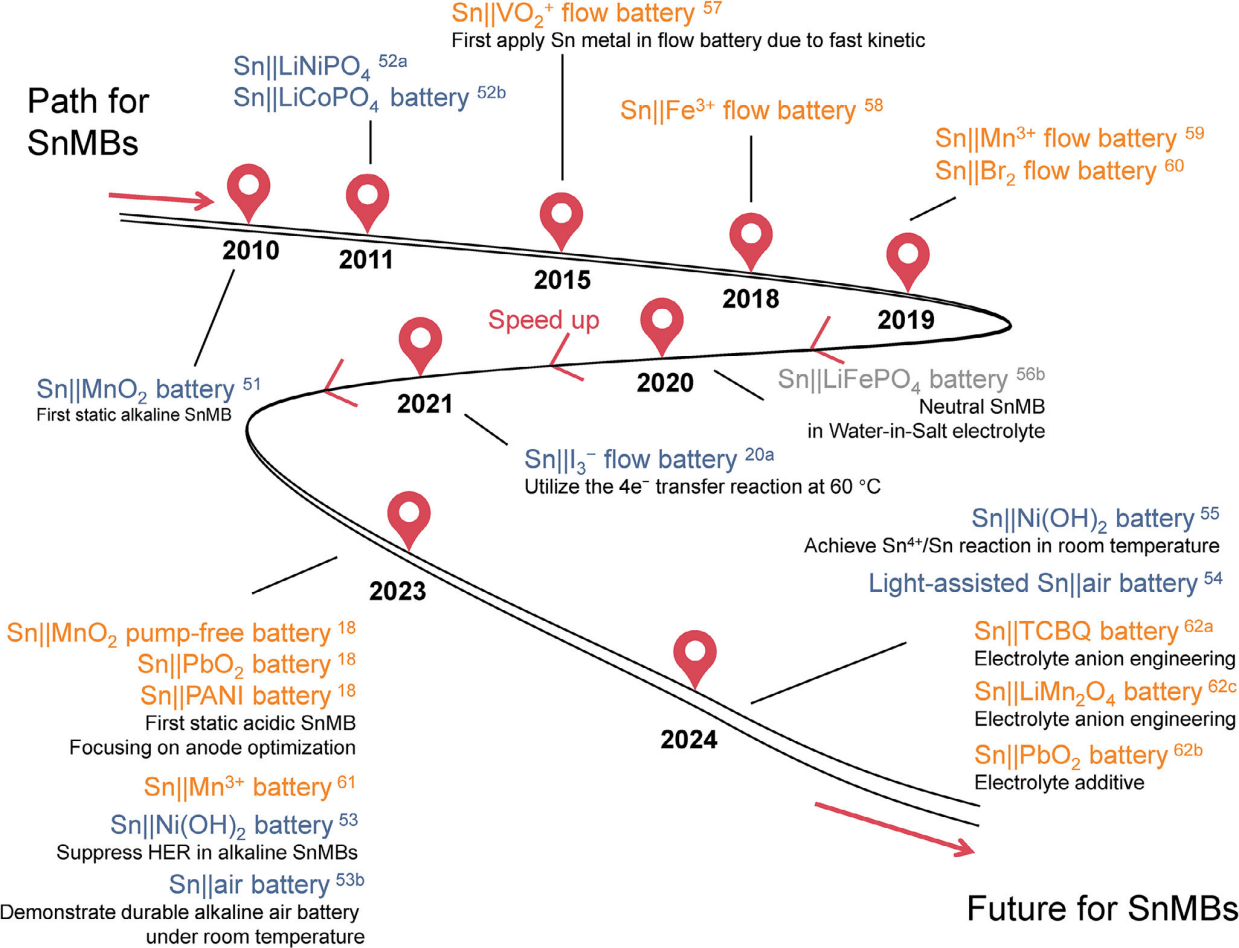
Roadmap scheme with typical achievements of Sn metal batteries. Blue means alkaline SnMB systems, grey means neutral SnMB systems, and red is acidic SnMB systems.^[^ ^18^, ^20^, ^51^, ^52^, ^53^, ^54^, ^55^, ^56^, ^57^, ^58^, ^59^, ^60^, ^61^, ^62^ ^]^

SnMBs in an aqueous alkaline system were first proposed in 2010 by Minakshi from Murdoch University, Australia, using γ‐MnO_2_ cathode and Sn metal anode in a saturated LiOH aqueous electrolyte (**Figure** **3**).^[^ ^51^ ^]^ This first reported alkaline SnMBs provided an output voltage of 1 V and a discharge capacity of 110 mAh g^−1^ with stability advantages over Zn under limited cycling performance (85% capacity retention after 50 cycles). Lithium‐intercalation/deintercalation compounds were applied as cathodes in LiOH‐based SnMBs afterward but the performance was still less than desirable.^[^ ^52^ ^]^ The lack of appropriate characterization tools limited the cognition, which researchers believed the Sn may convert to Li_4.4_Sn instead of Sn ions. Without Sn salts added to the electrolyte, the reversibility of Sn anode remains unsatisfactory. The study on static alkaline SnMBs was somewhat stalled until 2023 when two different research groups reported reversible 2e^−^ transfer Sn stripping/plating in KOH + Sn(OH)_3_^−^ electrolyte nearly simultaneously.^[^ ^53^ ^]^ Two types of batteries, Sn//Ni(OH)_2_ and Sn//air batteries were proposed with promising energy densities. These systems attracted instantaneous attentions in a short time for further enhancement investigations such as light‐assisted Sn//air batteries.^[^ ^54^ ^]^ Sn stripping/plating chemistry with 4e^−^ transfer from Sn^0^ to Sn^4+^ can provide more capacity but with a major challenge in the reversibility. Yao et al. first achieved the reversible Sn/Sn(OH)_6_^2−^ conversion in an aqueous redox flow battery under high temperature (60 °C) in 2021 and demonstrated better performance than Zn because Sn is less prone to dendrite growth.^[^ ^20a^ ^]^ Very recently, Mefford and co‐workers verified a 2‐step redox process of Sn/Sn(OH)_6_^2−^ at room temperature and achieved reversible Sn//Ni(OH)_2_ SnMBs based on four‐electron transfer mechanism (143.1 Wh L_cell_^−1^) by applied lean electrolyte and selective membrane.^[^ ^55^ ^]^ Such competitive performance to popular Zn metal batteries shows SnMBs as an alternative for high‐energy aqueous batteries.^[^ ^56^ ^]^

Fast kinetics, high capacity, and low redox potential render metallic Sn anode unique choices in acidic batteries. Sn was first applied in acid as an anode in aqueous redox flow batteries in 2015 due to its fast kinetics and stability under a current density of 180 mA cm^−2^.^[^ ^57^ ^]^ Sn metal has been since paired with a series of aqueous flow battery catholytes based on Fe^3+^,^[^ ^58^ ^]^ Mn^3+^,^[^ ^59^ ^]^ and Br_2_^[^ ^60^ ^]^ and delivered good energy efficiency. However, all these flow batteries used carbon‐based current collectors and did not optimize Sn‐based anolyte, resulting in subpar cycling performance. In 2023, Meng, Lu, and their co‐workers first focused on the Sn anode optimization and applied Sn metal as a universal anode in static aqueous acidic batteries, demonstrated a series of SnMBs with different cathode chemistries including Sn//MnO_2_ battery (stripping/plating), Sn//PbO_2_ battery (solid conversion) and Sn//PANI battery (de/intercalation).^[^ ^18^ ^]^ Sn//Mn^3+^ SnMB was also developed using proton and complexing agents to achieve a reversible Mn^3+^/Mn^2+^ cathode.^[^ ^61^ ^]^ Since then, more research strategies have developed to improve the reversibility of Sn metal anode in various acidic SnMBs.^[^ ^62^ ^]^ Emerging Sn metal anode studies initiated a major challenge to the traditional lead anode in energy, power, and sustainability. In addition, the advanced kinetic feature of Sn metal provides an excellent match to the proton battery cathodes of fast proton intercalation. Up to now, all existing acidic Sn anode chemistry is based on 2e^−^ transfer process. Although the redox potential gap between Sn(IV) species and Sn metal in acid is larger than that in alkaline, the study of reversible four‐electron Sn anode with high capacity may still represent another direction for further development of acidic SnMBs.^[^ ^63^ ^]^ Furthermore, while research on near‐neutral SnMBs battery systems remains limited, interest in these systems is steadily growing.^[^ ^56b^ ^]^ However, their performance faces significant competition from the well‐established zinc metal batteries in near‐neutral systems. Advancing the development of near‐neutral Sn metal batteries, especially through achieving a reversible four‐electron reaction, could significantly enhance their competitiveness and potential.

## 4 Growth Behavior and Challenge of Sn Metal Electrodeposition

To build reversible SnMBs, it is essential to understand the growth behavior of Sn metal. It is well known that Sn had four allotropes, including α‐, β‐, γ‐ and σ‐Sn. The latter two types of tin are only existing in high temperature (>161 °C) and high pressure (>2.9 GPa) conditions, which will not be detailly discussed here.^[^ ^64^ ^]^ Under atmospheric pressure, Sn metal mainly exists as nonmetallic α‐Sn and metallic β‐Sn, and their critical transition temperature is 13.2 °C.^[^ ^65^ ^]^ The most common form of Sn in practical applications is ductile metallic β‐Sn, which is stable at room temperature and above. Before its use in aqueous batteries, β‐Sn is extensively utilized as an anode material in non‐aqueous battery systems, including lithium‐ion and sodium‐ion batteries.^[^ ^66^ ^]^ The low‐temperature phase of Sn, known as α‐Sn or gray tin, adopts a diamond cubic structure similar to that of diamond and silicon.^[^ ^67^ ^]^ The atoms in α‐Sn form a covalent structure, restricting free electron movement and resulting in nonmetallic properties, making it unsuitable for electrodeposition. Below the critical transition temperature, β‐Sn gradually but spontaneously transforms into dark gray α‐Sn powder, a phenomenon commonly referred to as “tin pest”. This feature needs the researchers to become super careful when designing low‐temperature SnMBs. Fortunately, the phase transition process can be suppressed by various strategies like doping Sb and Bi to stabilized the crystal structure of Sn.^[^ ^68^ ^]^

**Figure 4:**
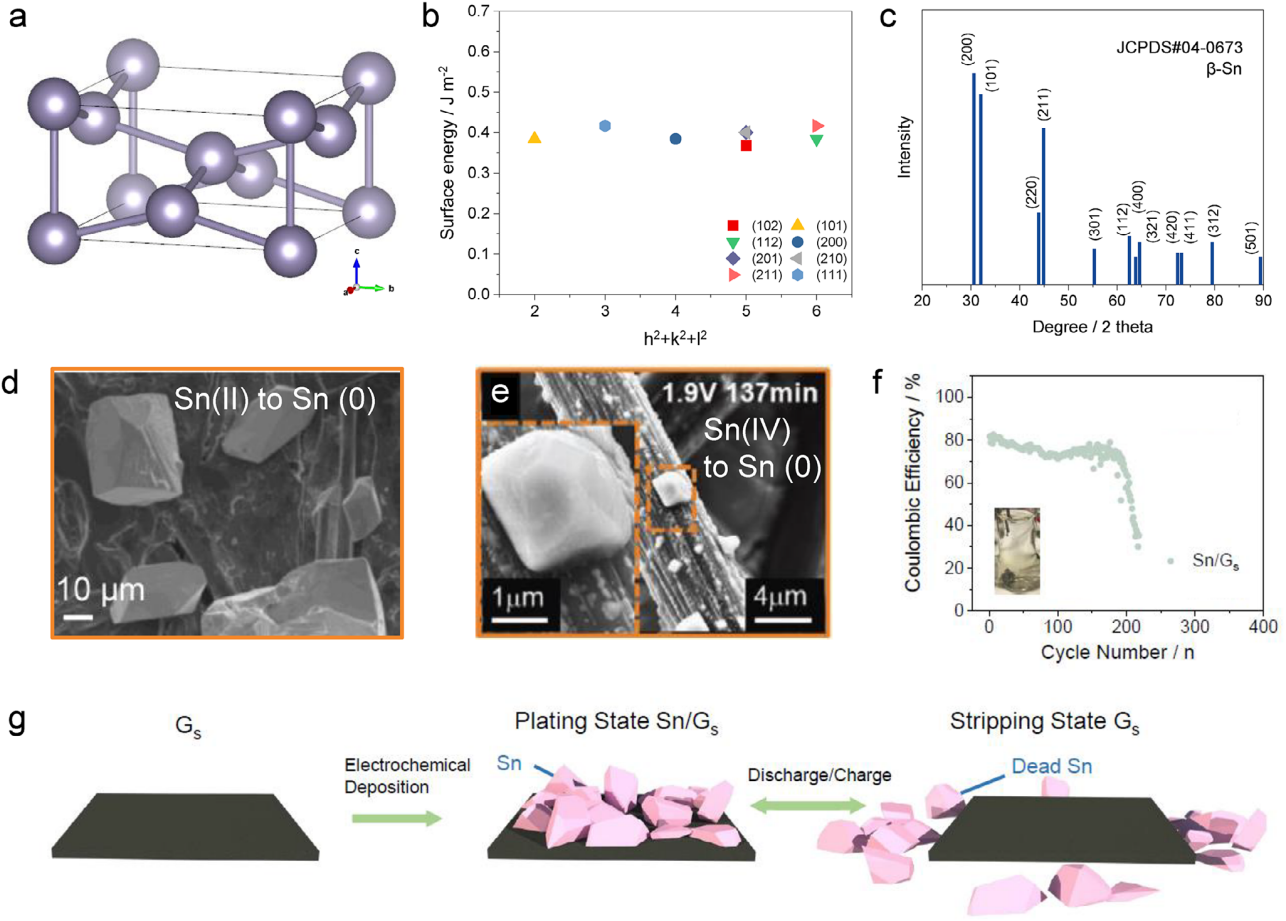
a) The crystal structure of β‐Sn (JCPDS#04‐0673). b) Reported surface energies of different lattice planes in Sn metal.^[^ ^20b^ ^]^ c) Theoretical standard X‐ray diffraction pattern of Sn metal. d) SEM image for Sn deposition on carbon substrate from Sn(II) salt. Reproduced with permission.^[^ ^18^ ^]^ Copyright 2023, Elsevier. e) SEM image for Sn deposition on carbon substrate from Sn(IV) salt. Reproduced with permission.^[^ ^20a^ ^]^ Copyright 2021, Wiley‐VCH. f) Coulombic efficiency of Sn on carbon substrate in acidic electrolyte with a fixed charge capacity of 1 mAh cm^−2^ at 2 mA cm^−2^. Inside are optical photographs after the cycling test. g) Schematic illustration of the Sn stripping/plating chemistry on a carbon substrate. Reproduced with permission.^[^ ^18^ ^]^ Copyright 2023, Elsevier.

β‐Sn has a body‐centered tetragonal (BCT) crystal structure (**Figure** **4**a), which is different from most commonly used metal anodes like body‐centered cubic (BCC) type metals (e.g., Li, Na), face‐centered cubic (FCC) type metals (e.g., Al, K) and hexagonal close‐packed (HCP) type metals (e.g., Mg, Zn).^[^ ^69^ ^]^ The lattice is stretched along one axis when creating the tetragonal structure, which means the atoms may arranged in a way that results in a more uniform distribution of energy across different lattice planes depending on the c/a ratio. This elongation can reduce the anisotropy of surface energy between different planes, which may leading to more similar surface energies in different crystallographic directions compared to other structures like BCC and HCP.^[^ ^70^ ^]^ As shown in Figure 4b, β‐Sn shows very similar surface energy among different lattice planes by DFT calculations,^[^ ^20b^ ^]^ and the standard X‐ray diffraction pattern shows at least 3 strongest peaks with similar intensities (Figure 4c). Sn metal exhibits a propensity to form polyhedral particles during electrodeposition, favoring isotropic growth over the development of films or dendritic structures. This tendency has been substantiated by multiple studies, which have demonstrated that Sn readily forms polyhedral particles under both acidic (Figure 4d)^[^ ^18^ ^]^ and alkaline electrodeposition conditions (Figure 4e).^[^ ^20a^ ^]^ The crystal growth behavior of Sn during deposition indeed mitigates the risk of puncturing the separator and causing short circuits, unlike the dendritic structures typically formed by Li and Zn.^[^ ^71^ ^]^ However, when the particles become too large (tens to hundreds of microns), the edges and corners could still damage the separator for potential short circuiting. The non‐uniform Sn particles can also compromise their contact with the current collector, leading to possible detachment to form electrochemically inactive Sn. Figure 4f shows the cycling performance of the Sn metal anode deposited on the graphite current collector with the same condition in Figure 4d. The graphite substrate is used without any specific surface treatment, and the electrolyte employed is a standard composition commonly used in the electrodeposition industry, consisting of 2 M H_2_SO_4_ and 0.1 M SnSO_4_. Under these conditions, the Sn anode demonstrates a Coulombic efficiency of less than 80% and fails within 200 cycles. Post‐cycling analysis reveals the presence of unconnected Sn particles at the bottom of the cell, identified as electrochemically inactive “dead Sn”, analogous to the concept of “dead Li” observed in Li metal systems.^[^ ^72^ ^]^

One representative degradation mechanism is illustrated in Figure 4g. Sn tends to grow along multiple crystal faces to form particles, resulting in uneven electric field distribution on the electrode surface. The free diffusion of Sn ions leads to their accumulation at protrusions due to the high local charge density, driving reductive growth and the formation of larger particles. These larger particles exhibit limited contact with the current collector, making them prone to detachment and loss of electrical connectivity. More importantly, according to the time scale of electron transport τ≈L^2^/D, Sn particles preferentially dissolve from the bottom during the stripping process, leading to their fragmentation and eventual detachment from the electrode, resulting in the formation of “dead Sn”.^[^ ^73^ ^]^ Meanwhile, the HER may also be accelerated due to extensive interfacial area between the electrolyte and Sn particles, further deteriorating the adhesion and electrical connections between the Sn particles and the substrate. In addition to the intrinsic stability of Sn metal, side reactions such as HER can significantly reduce the Coulombic efficiency, consuming the electrolyte, and potentially leading to safety hazards like explosions. Although HER kinetics on Sn are inherently sluggish, this competing side reaction may still occur during long‐term cycling or under conditions of high current density and high capacity, particularly in acidic environments. The dynamic degradation of Sn ions in the electrolyte is another critical issue to consider. Sn can exist in the electrolyte as either divalent or tetravalent ions, and their interconversion under varying external conditions can result in undesirable electrolyte degradation and increased risk of self‐discharge.^[^ ^55^, ^62^ ^]^ Mitigating this transformation is crucial for preventing electrolyte attenuation and achieving reversible Sn anode. Consequently “dead Sn” issue, HER side reaction, and self‐degradation could be identified as the main critical issues for the Sn metal anode. Addressing these challenges is essential for the development of high‐performance SnMBs.

## 5 Strategies for Optimizing Sn Metal Anodes

Although the development of SnMBs is still in the incipient stage, several strategies have recently been proposed to optimize Sn metal anodes. The scope includes interfacial engineering of the Sn metal, electrolyte development, and separator design aimed at regulating metal deposition and mitigating side reactions. These approaches aim to suppress hydrogen evolution and mechanical instability, significantly enhancing the cycling performance and stability of SnMBs. A comprehensive understanding of these optimization strategies, coupled with an in‐depth analysis of the structure–performance relationship, will be explored in the following sections. Meanwhile, the performance evaluation of Sn anode optimized by different approaches is selected in Table 1. This summary aims to offer valuable insights to guide the advancement of Sn metal anode designs for high‐performance energy storage systems.

**Table 1:** The electrochemical performance of selected Sn metal anodes in published works.^[^ ^18^, ^53^, ^55^, ^56^, ^61^, ^62^ ^]^

Anode materials	Strategy	Electrolyte	Coulombic Efficiency [%/mAh cm^−2^]	Cycling performance (symmetric cell) [hours/current density [mA cm^−2^]/capacity [mAh cm^−2^]	Cycling performance (asymmetric cell) [%CE/cycles/current density [mA cm^−2^]/capacity [mAh cm^−2^]	Refs.
Sn/Cu	Interfacial engineering	2 M H_2_SO_4_ + 0.1 M SnSO_4_	98/1	N/A	91/350/2/1	[18]
Sn/Cu	Interfacial engineering/Electrolyte development	3 M MSA + 1 M Sn(MSA)_2_	99.95/1	N/A	99.95/1700/10/1	[62a]
Sn/graphite paper	Electrolyte development	2 M H_2_SO_4_ + 0.2 M SnSO_4_ + 1% POPE	99/1	N/A	92/1300/6/1	[62b]
Sn foil	Electrolyte development	1 M SnCl_2_	99.97/1	2780/0.5/0.25	99.97/1532/1/1	[62c]
Sn plate	N/A	3 M H_2_SO_4_ + 0.1 M SnSO_4_	N/A	560/0.5/0.25	N/A	[61]
Sn powder	Electrolyte development	20 M LiTFSI + 1 M Sn(OTf)_2_	95/0.075	330/0.2/0.033	N/A	[56b]
Sn/brass mesh	Interfacial engineering	6 M KOH + 0.2 M Sn(OH)_3_^‒^ (from SnO)	99.9/0.5	N/A	∼100/2750/10/1	[53b]
Cu@Sn	Interfacial engineering	3 M KOH + 0.1 M SnSO_4_	87.8/0.5	560/10/0.5	82.3/180/10/0.5	[53a]
Sn/graphite felt	Separator design	0.86 M KOH + 1.7 M K_2_Sn(OH)_6_	99/2	N/A	N/A	[55]

### 5.1 Interfacial Engineering of Sn Metal

The electroplating process of Sn metal at the anode–electrolyte interface involves several sequential stages, including Sn ion diffusion through the electrolyte, electrochemical reduction of Sn, nucleation of Sn atoms, and subsequent crystal growth.^[^ ^74^ ^]^ Each of these steps is associated with an energy barrier that governs the efficiency and morphology of the Sn deposition.^[^ ^24a^ ^]^ To promote uniform deposition and suppress ununiform particle formation, it is crucial to ensure that the energy barrier for nucleation and growth is kept low and consistent throughout the electroplating process.^[^ ^75^ ^]^ Careful control of these parameters facilitates homogeneous Sn crystallization, leading to improved electrochemical performance and enhanced cycling stability of Sn‐based anodes. The nucleation and growth phases, in particular, are highly sensitive to the electroplating conditions, such as substrate surface topography, the uniformity of the electric field across the anode–electrolyte boundary, and the interfacial adhesion between the substrate and Sn.^[^ ^76^ ^]^ In the special case of Sn metal, the particle morphology in the most common scenario leads to a high risk of loose electrochemical connection, therefore an interface or interphase that can provide extra interaction will be benefit for suppress dead Sn formation.

**Figure 5:**
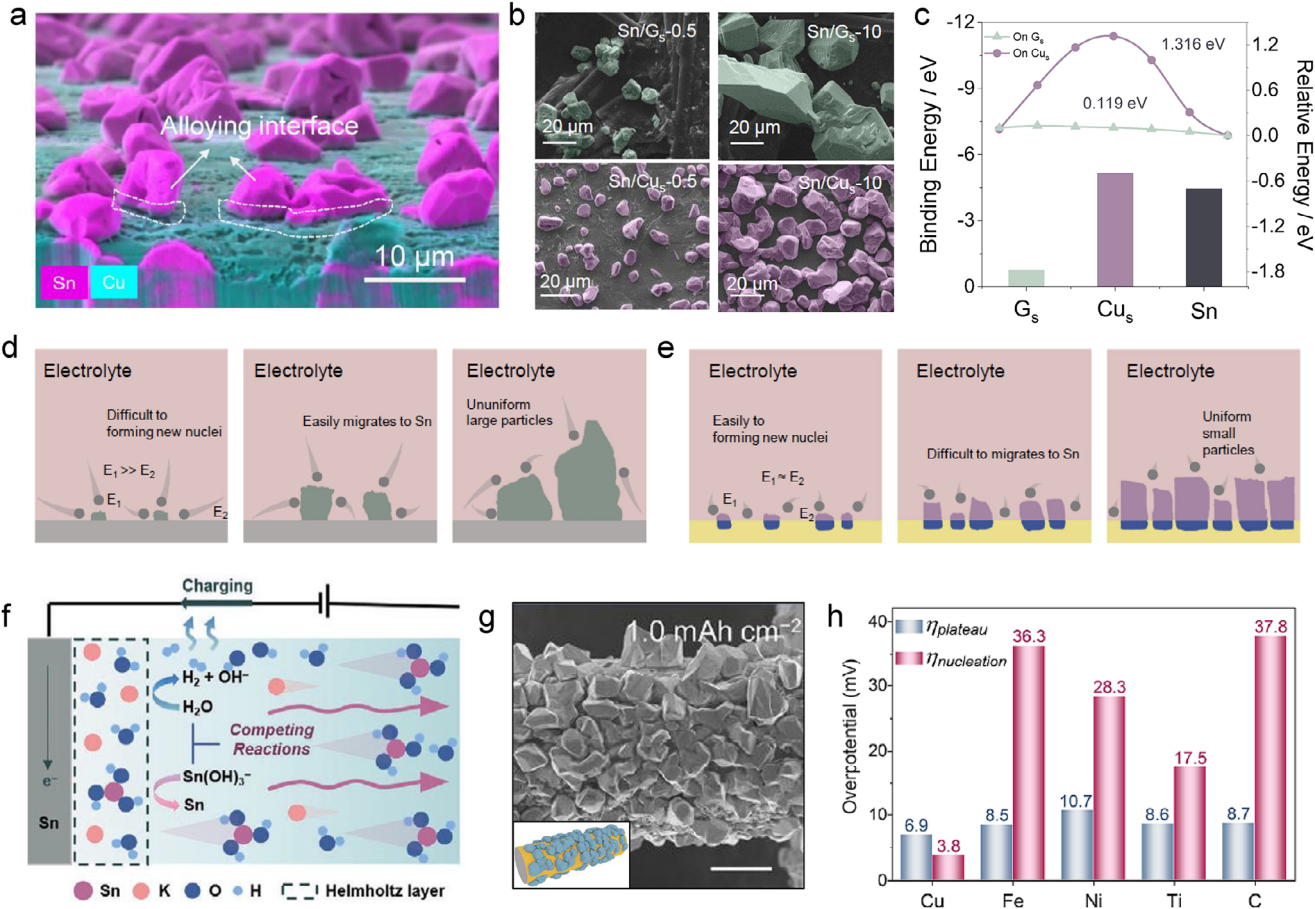
a) Side‐view SEM image and corresponding EDS mapping of Sn on Cu substrate at 0.5 mAh cm^−2^ focusing on the interface of Sn and Cu. b) SEM images for Sn deposition on carbon and Cu substrates with deposition capacities of 0.5 and 10 mAh cm^−2^. c) Theoretical calculated binding energy (bottom) and diffusion energy barriers (upper) of Sn^2+^ on graphite, copper, and tin surface. Illustration of the Sn metal deposition process on carbon d) and copper e) substrate. The blue area means the interfacial alloying interaction. E_1_ and E_2_ mean the absolute value of binding energy on Sn seed and substrate. Reproduced with permission.^[^ ^18^ ^]^ Copyright 2023, Elsevier. f) The plating mechanism on the Sn foil in alkaline which shows the hydrogen competing reaction. The arrows indicate the direction of Sn(OH)_3_^−^ diffusion. Reproduced with permission.^[^ ^53a^ ^]^ Copyright 2023, Wiley‐VCH. g) SEM images for Sn deposition on Cu substrates in alkaline electrolyte. h) Nucleation and plateau overpotentials on different substrates. Reproduced with permission.^[^ ^53b^ ^]^ Copyright 2023, American Chemical Society.

Sn has a high affinity to most of the metals (e.g., Pb, Cu, Fe, Bi, Cd) and easily forms binary alloys.^[^ ^77^ ^]^ When these heterogeneous metals serve as substrates, they not only enhance the interaction with deposited Sn particles through interfacial alloying but also can serve as nucleation seeds, reducing the energy barrier for Sn nucleation since they possess both good Sn affinity and high electronic conductivity. Besides, choosing appropriate metal substrates needs to evaluate stability in electrolyte, intrinsic HER activity to avoid extra side reactions, sustainability, and cost. Zhang et al. first applied copper foil as a substrate to regulate Sn metal deposition in an acid environment, improving the Coulombic efficiency from 78% to an optimized 98% compared to the traditional graphite substrate.^[^ ^18^ ^]^ The Cu substrate forms an alloying interphase with a thickness of ≈0.5 µm, and the interphase will not keep growth when the deposition capacity increases (**Figure** **5**a). Alloying interphase also results in some residual Sn nuclei after the first few cycles, which makes the following nucleation process easier and reflected in the reduced overpotential. Besides, the deposited Sn particles on the Cu substrate were smaller in size and more uniformly distributed compared to those on the graphite substrate (Figure 5b). Based on theoretical calculation results (Figure 5c), the deposition mechanism is concluded below. On graphite substrates (GS, Figure 5d), Sn atoms exhibit a preference for aggregating on existing nuclei rather than initiating new ones. This tendency arises because Sn has a higher binding energy to itself than to GS, leading to the formation of larger, uneven particles. Conversely, copper substrates (CuS, Figure 5e) offer a higher binding energy with Sn ions, which promotes a uniform electric field distribution and increases nucleation sites. Additionally, CuS presents a higher diffusion energy barrier (1.32 eV) compared to GS (0.12 eV), hindering the migration of Sn ions to already‐deposited Sn particles. This characteristic facilitates uniform deposition, resulting in smaller and more evenly distributed Sn particles. As a result, both interphase interaction and deposition behavior optimization contribute to better stability of Sn anode. Copper is considered the suitable candidate in following acidic SnMB works up to now due to the instability of other traditional metal substrates like Ni, Ti, and stainless steel in acid, but more choices still remain undeveloped.^[^ ^62a^ ^]^

In alkaline conditions, the Sn ion represents stannate anions that hold the same negative charge with the anode during the plating, with existing strong electrostatic repulsion force between the charge carriers and the electrode surface. It will be more difficult for stannate anions to reach the anode for proper deposition and might initiate severe competitive hydrogen evolution side reactions (Figure 5f). To solve this problem, brass substrates were used by Chao et al. to deliver reversible Sn(OH)_3_^−^/Sn chemistry in alkaline conditions, showing superior Coulombic efficiency and cycling performance compared to other substrates like carbon, Ni, Fe, and Ti.^[^ ^53b^ ^]^ Rock‐like Sn particles are distributed uniformly on the brass substrates at 1 mAh cm^−2^, and no dendrite was observed due to the isotropic feature of Sn (Figure 5g). In this situation, nucleation overpotential is attributed to the absorption of Sn(OH)_3_^−^, Sn─O bond breaking, and Sn nucleation on the substrate. With a low nucleation overpotential (<5 mV, Figure 5h), Cu facilitates efficient Sn nucleation due to strong chemisorption and Sn–Cu alloy formation. This tinophilic behavior ensures strong adhesion of Sn particles, promoting uniform deposition and improved battery performance. Lu and his co‐workers also introduced a nondense copper layer on Sn metal foil via electrodeposition, which is able to facilitate the adsorption behaviors of Sn(OH)_3_^−^ ion, suppress the metal shedding, and alleviate the HER competitive reaction.^[^ ^53a^ ^]^ As a result, the efficiency of the Sn redox reaction in alkaline electrolyte increased fivefold to ≈100% compared to bare Sn metal foil and can be cycled more than 560 h at 0.5 mAh cm^−2^. These works show that efficient interfacial engineering could regulate the stripping/plating process for Sn metal, thus improving the reversibility. Very recently, Fan et al. developed a stannophilic Ag‐coated vertical graphene substrate for uniform Sn metal deposition. Acid‐tolerant Ag sites improve the nucleation kinetics near the interface, enable reversible Sn stripping/plating, and suppress the formation of dead Sn. This strategy combining interfacial engineering and 3D substrate design inspires the Sn deposition regulation, especially at high areal capacities.^[^ ^78^ ^]^

### 5.2 Electrolyte Development

In SnMBs, the electrolyte is a crucial component, not only facilitating ion transport between the anode and cathode but also directly influencing the electrochemical stability, efficiency, and longevity of a battery.^[^ ^79^ ^]^ The composition of the electrolyte governs key processes such as ion diffusion, surface reactions at the electrodes, and the suppression of undesirable side reactions. Additionally, the electrolyte plays a vital role in maintaining the structural integrity of the Sn anode, which is prone to the “dead Sn” issue. Proper electrolyte engineering in aqueous SnMBs can significantly stabilize the anode‐electrolyte interface and improve the Coulombic efficiency of Sn redox reactions. Strategies like optimizing the ionic composition and introducing additives have proven effective in regulating ion transport, minimizing parasitic reactions, and promoting uniform Sn deposition. Tailoring electrolyte properties is essential for ensuring long‐term battery performance.

**Figure 6:**
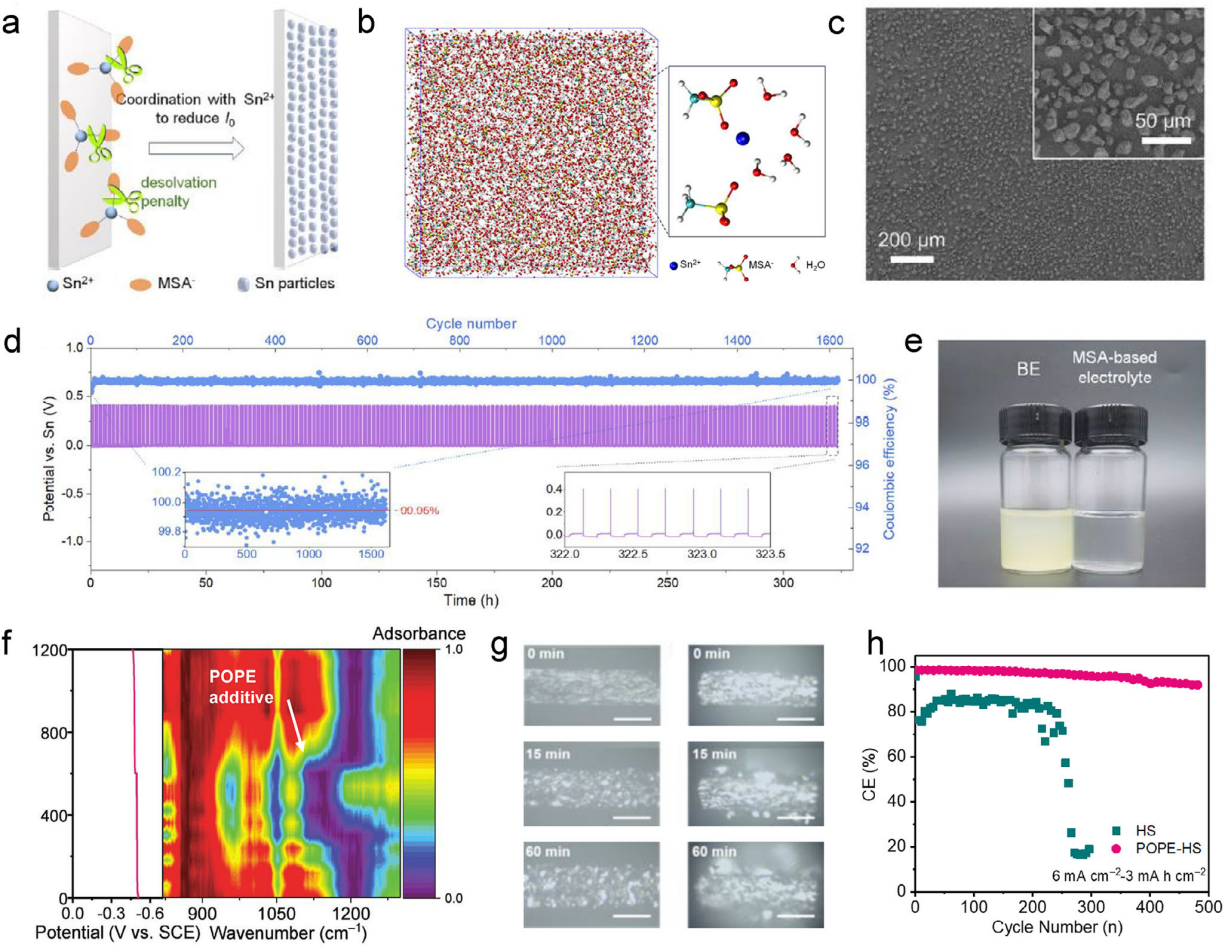
a) The adsorption layer formed by GEL can slow the Sn plating kinetics, leading to uniform deposition. b) Snapshot illustration of the MD simulation cell and Sn^2+^ solvation structure in MSA‐based electrolyte. c) SEM images of the Sn deposition morphology in the MSA‐based electrolyte with a capacity of 1 mAh cm^−2^. d) Electrochemical Sn plating/stripping behavior in the MSA‐based electrolyte at a current density of 10 mA cm^−2^ with 1 mAh cm^−2^. e) Photograph of H_2_SO_4_‐based after quiescence for 1 week and the MSA‐based electrolyte after quiescence for 1 month. Reproduced with permission.^[^ ^62a^ ^]^ Copyright 2024, Elsevier. f) The in situ FT‐IR spectroscopy of the Sn plating/stripping process in POPE added acidic electrolyte. g) In situ optical photographs of Sn deposition in (left) POPE added electrolyte and (right) regular acidic electrolytes after different deposition times at 20 mA cm^‒2^ (scale bar: 50 µm). h) Coulombic efficiency of the Sn stripping/plating in sulfuric acid‐based electrolytes at 6 mA cm^‒2^ with the capacity of 3 mAh cm^‒2^. Reproduced with permission.^[^ ^62b^ ^]^ Copyright 2024, Wiley‐VCH.

In acidic and alkaline conditions, the Sn ions are surrounded and complexed by the species from the electrolyte and form solvation sheaths. Different electrolyte compositions can modulate the solvation sheaths of Sn ions and control the deposition or side reaction. Yu et al. replaced traditional sulfuric anions with methylsulfonic anions (MSA) in acidic electrolyte to regulate the complexing environment of Sn^2+^ because of its decent coordination interaction with metallic ions (**Figure** **6**a).^[^ ^62a^ ^]^ Compared with bisulfate anions, MSA anions have higher energy levels of the highest occupied molecular orbital (E_HOMO_) and are easier to donating electrons, thus benefit to interact with Sn^2+^ cations. The simulation results show that MSA anion can be involved in the first solvation shell with a coordination number of 2.6 (Figure 6b). As a result, the increasing complexing interaction between Sn cation and MSA anion increases the desolvation penalty of the complex during the deposition, leading to a lower exchange current density and improved nucleation behavior. SEM images of Sn deposited from MSA‐based electrolyte in Figure 6c exhibit small and uniform distributed Sn particles, leading to durable cycling performance of more than 300 h at 1 mAh cm^−2^ and high Coulombic efficiency (99.95%, Figure 6d). For SnMB electrolyte with 2e^−^ transfer chemistry, the Sn(II) in the electrolyte is easily oxidized to Sn(IV) when contacting the oxygen, leading to the failure of the system. In this scenario, an MSA‐based electrolyte holds another advantage preventing the oxidation of Sn^2^⁺ to Sn⁴⁺ due to its reducing nature over a month (Figure 6e), which is important for avoiding self‐degradation of SnMBs during fabrication and daily use. Except for the MSA anion, the Cl^−^ anion was also studied in an acidic system to replace sulfuric acid. The SnCl_2_ electrolyte applied by Chang et al. does not undergo hydrolysis based on its Cl^−^ complex ligands and provide a low acidic pH of 1.09.^[^ ^62c^ ^]^ This low acidic condition weakened the tendency of hydrogen evolution and resulted in good reversibility.

Electrolyte additive is a general method to lower nucleation barrier, improve reversibility, mitigate side reactions, regulate deposition morphology, etc. by forming solid electrolyte interphase (SEI) or controlling the species structure near the interface.^[^ ^80^ ^]^ However, SEI is hard to form by the electrolyte additive in the acid because of the strong corrosion of the proton, but the solvation structure can still be induced at the interface. For instance, Xu and Zhang et al. applied 4‐tert‐octylphenol pentaethoxylate (POPE), a cheap and non‐toxic chemical product as an effective additive to in situ modify the electrode/electrolyte interface based on interfacial coordination.^[^ ^62b^ ^]^ POPE is rich in polar hydroxyl (O‐H) and ether (C‐O‐C) functional groups to selectively coordinate with Sn. A POPE adsorbed layer can be in situ formed on the Sn surface (Figure 6f) to screen from the acid medium and give more inhibition to the parasitic HER, facilitating an even electric field distribution and uniform Sn deposition/dissolution process (Figure 6g). As a result, the Sn foil electrode was able to maintain more than 91.9% Coulombic efficiency after being cycled 500 times at 3 mAh cm^−2^ with the existing POPE additive, but the Sn anode in non‐additive electrolyte only held a Coulombic efficiency less than 80% at the beginning and dramatically failed after 200 cycles (Figure 6h). Besides, the gelatin was also employed as an additive to adsorb on the electrode surface, forming an adsorption layer and slowing down the Sn plating kinetics for more uniform deposition. Only 1.0 g L^−1^ gelatin could effectively decrease the current density of Sn deposition from 9.16 to 3.55 mA cm^−2^, leading to a uniform Sn particle distribution.^[^ ^62a^ ^]^ Although electrolyte engineering is already widely applied in various mature battery systems as one of the most efficient strategies, the relevant application in aqueous SnMBs is still under development, especially for alkaline systems. The appropriate electrolyte optimization could play an important role in reversibility, power density, and even energy density for SnMBs.

### 5.3 Separator Design

**Figure 7:**
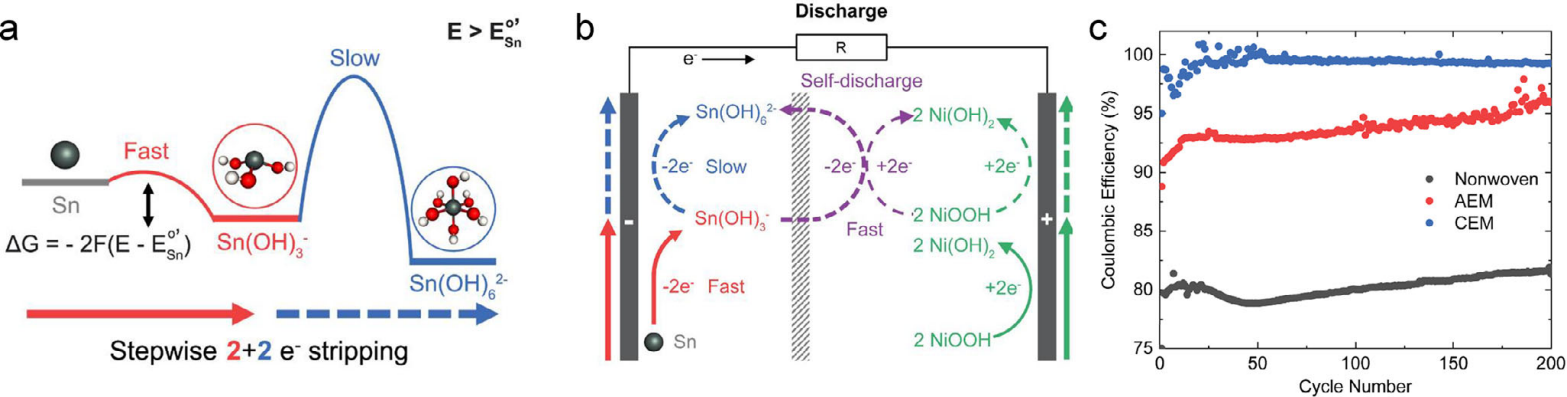
a) Proposed mechanisms of the Sn stripping process for a four‐electron stepwise 2 + 2 e^−^ stripping resulting from the rate disparities in successive electron transfers. b) Mechanism illustration of Ni(OH)_2_//Sn battery based on four‐electron battery. Where the purple line shows the shuttle effect of the soluble Sn(OH)_3_^−^ intermediate induces self‐discharge. c) A comparison of Coulombic efficiencies for cells utilizing various separators during extended cycling tests. Reproduced with permission.^[^ ^55^ ^]^ Copyright 2024, Elsevier.

Separators in SnMBs play a crucial role in preventing physical contact between the electrodes while allowing ion transport. An ideal separator should exhibit excellent ionic conductivity, moderate porosity, and strong wettability with the electrolyte to facilitate efficient ion exchange. While commonly used separators, such as glass fiber, filter paper, and polypropylene, have limited impact on overall cell performance, applying surface functionalized separator or selective ion exchange membrane separators offer promising strategies for enhancing electrochemical stability and achieving improved battery performance.^[^ ^81^ ^]^ Recently, Mefford and his co‐workers verified that the separator selection plays a critical role in achieving reversible four‐electron Sn anode chemistry in Ni(OH)_2_//Sn SnMB system.^[^ ^55^ ^]^ Using electrochemical quartz crystal microbalance with the rotating ring disk electrode, the researchers found that the plating of Sn from KOH + K_2_Sn(OH)_6_ alkaline electrolyte is a one‐step reaction directly reduced from Sn(OH)_6_^2−^ to Sn metal. During the stripping process, Sn is preferentially oxidized to the two‐electron soluble product Sn(OH)_3_^−^ first instead of Sn(OH)_6_^2−^, then the diluted Sn(OH)_3_^−^ may gradually oxidized to higher valance resulting in a stepwise 2 + 2 e^−^ process (**Figure** **7**a). This asymmetric plating/stripping behavior could be attributed to the sluggish kinetics of the Sn(OH)_6_^2−^/Sn(OH)_3_^−^ couple compared with Sn(OH)_3_^−^/Sn. A lean electrolyte cell was applied to restrict the redox intermediate Sn(OH)_3_^−^ near the electrode to obtain reasonable Coulombic efficiency. However, the soluble Sn(OH)_3_^−^ intermediate from the four‐electron Sn redox reaction may easily migrate through the traditional separator and can be chemically oxidized by the cathode like NiOOH with more positive operational potential, which significantly limits the Coulombic efficiency due to self‐discharge (Figure 7b). An ion exchange membrane was used as a separator to limit the migration of discharge intermediates. A cation exchange membrane (CEM) can restrict the transport of Sn(OH)_3_^−^ anions, while an anion exchange membrane (AEM) may also hinder Sn(OH)_3_^−^ transport but selectively allows OH⁻ ions to pass due to size differences. Replacing the separator with ion‐selective membranes significantly reduced the permeability of Sn(OH)_3_^−^ anions, leading to a substantial increase in Coulombic efficiency—rising from 80% without a membrane to 92% with an AEM and 99% with a CEM (Figure 7c). The ion exchange membrane separator is also employed in other alkaline SnMB systems to prevent the crossover of intermediates and could potentially be applied to the emerging four‐electron Sn chemistry in acidic environments as well.

## 6 Progress and Potential of Sn Metal Batteries

Up to now, significant progress has been made in establishing preliminary, cyclable Sn metal anodes (1–3 mAh cm^−2^, 99% Coulombic efficiency, hundreds of cycling hours) through the dedicated efforts of researchers. Based on these advancements, a variety of Sn metal batteries have gradually been developed. In this section, we systematically analyze the strengths and weaknesses of current typical Sn metal‐based full cell systems and explore the potential of emerging SnMB technologies based on the pH of the electrolyte (acidic and alkaline system). The goal is to harness the advantages of Sn anodes to develop efficient, low‐cost, and sustainable energy storage solutions.

### 6.1 Acidic Sn Metal Batteries

Aqueous acidic batteries society has been dominated by lead‐acid batteries in the past 150 years since Gaston Plante invented the protype for lead‐acid batteries. The total energy scale of lead‐acid batteries has reached terawatt‐hour (TWh) level and still holds 35% batteries market under the sharp development of Li‐ion batteries.^[^ ^83^ ^]^ Lead‐acid battery can provide an output voltage of 2 V, a theoretical capacity of 120 mAh g^−1^ based on cathode and anode, and a practical cell‐level energy density of 30–50 Wh kg^−1^.^[^ ^84^ ^]^ The reasonable energy provided by the lead‐acid battery is benefiting from low redox potential (−0.36 V vs SHE) and relatively high capacity of Pb metal (259 mAh g^−1^). However, the lead anode suffered from bad electron conductivity of its discharge product PbSO_4,_ and the sluggish solid‐phase transforms kinetics, so the capacity utilization is usually less than 50% and the cycling stability is unsatisfactory.^[^ ^85^ ^]^ Nevertheless, the lack of suitable anodes in the corrosive acidic environment with a large number of protons has forced a considerable part of the current development of new acidic batteries to use lead anodes. From a sustainability perspective, continuing to develop lead‐based battery systems is unsuitable due to the high toxicity of lead to the environment and human health.

**Figure 8:**
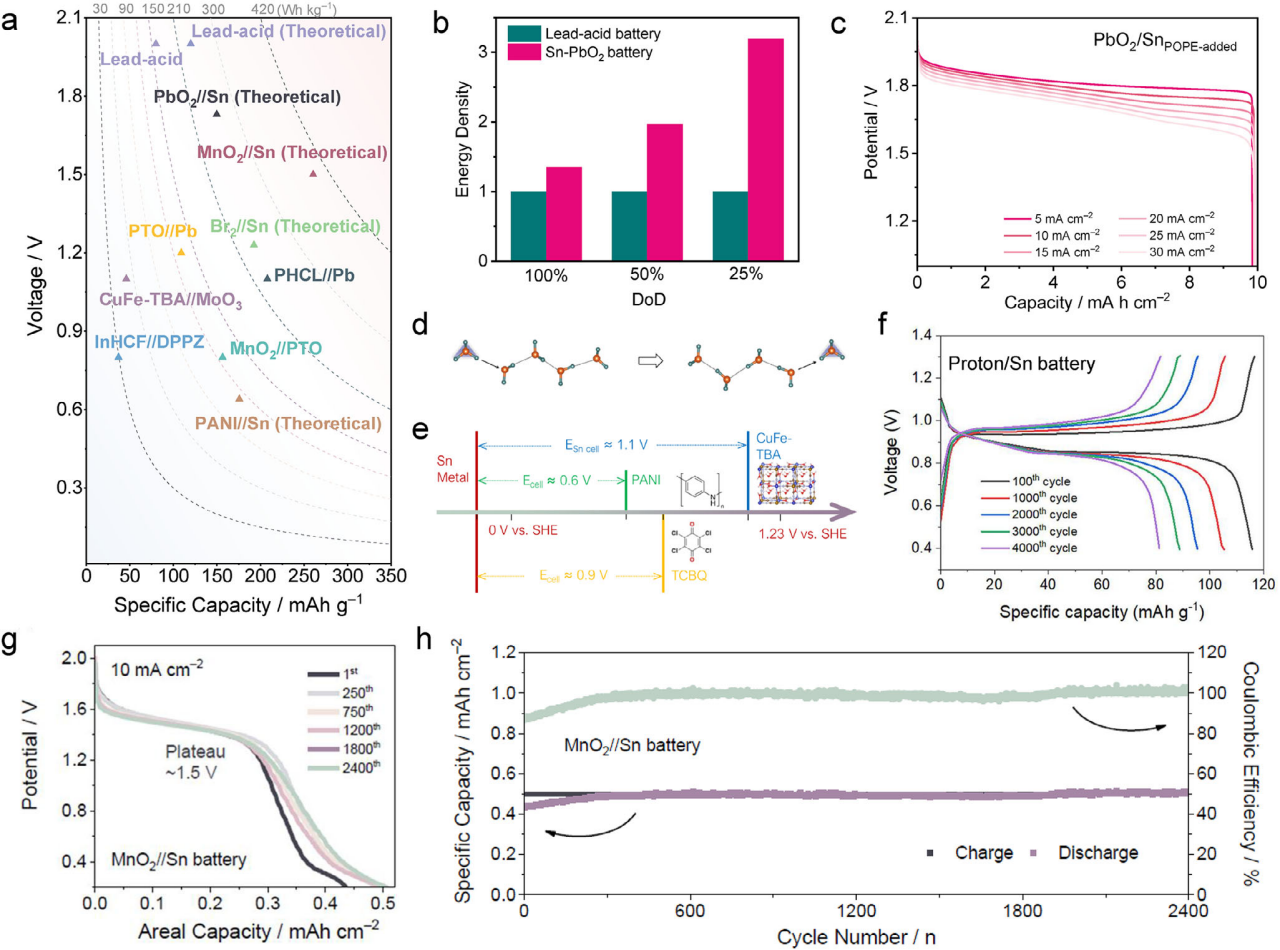
a) Cell voltage, specific capacity, and energy density of typical SnMBs compared with other aqueous acidic batteries based on cathode and anode mass loading.^[^ ^3^, ^49^ ^]^ b) The calculated energy densities comparison between PbO_2_//Sn battery and lead‐acid battery at different DoD of the anodes. c) GCD curves of PbO_2_//Sn battery at different current densities with a charge capacity of 10 mAh cm^‒2^ with POPE electrolyte additive. Reproduced with permission.^[^ ^62b^ ^]^ Copyright 2024, Wiley‐VCH. d) Grotthuss mechanism in proton transfer. Reproduced with permission.^[^ ^82^ ^]^ Copyright 2021, Wiley‐VCH. e) Voltages of recently reported proton battery cathodes and theoretical voltages of proton//Sn batteries. f) Typical charge/discharge curves of the TCBQ//Sn batteries at 6 A g^‒1^ with proton battery cathode. Reproduced with permission.^[^ ^62a^ ^]^ Copyright 2024, Elsevier. g) Discharge curves of pump‐free MnO_2_//Sn battery at different cycles with a fixed charged capacity of 0.5 mAh cm^‒2^ at 10 mA cm^‒2^. h) Long‐term cycling performance at 10 mA cm^‒2^. Reproduced with permission.^[^ ^18^ ^]^ Copyright 2023, Elsevier.

The appearance of Sn metal holds a unique position due to its performance and sustainability advantage compared with Pb. The wide pH operating range and high acid tolerance of Sn allow it to be compatible with a broad range of cathodes and deliver exciting performance (**Figure** **8**a).^[^ ^3^, ^49^ ^]^ Although the redox potential of Sn^2+^/Sn is 0.22 V higher than Pb, high specific capacity and potentially high utilization rate of stripping/plating Sn chemistry could well compensate for the negative impact of voltage decline and provide more competitive energy density. When replacing Pb with Sn in lead‐acid batteries, a PbO_2_//Sn battery can provide 35% higher theoretical energy density compared to traditional lead‐acid batteries at 100% depth of discharge (DOD) for both anodes (based on cathode and anode, Figure 8b).^[^ ^62b^ ^]^ In practical applications, lead anodes typically operate at a maximum of 50% DOD to ensure satisfactory cycle stability, while Sn can still cycle stably at ≈100% DOD (based on the anode). This increases the theoretical energy density advantage of PbO_2_//Sn batteries to almost twice that of lead‐acid batteries. Although the difference in cell‐level energy density may be diminished in practical manufacturing scenarios, and lead‐acid battery designs have long been optimized for commercialization, this nonetheless underscores the promising potential of PbO_2_//Sn batteries. As shown in Figure 8c, the lead dioxide‐tin batteries demonstrate a flat discharge plateau ≈1.8 V and can operate stably at currents of 5–30 mA cm^‒2^ with a capacity of 10 mAh cm^‒2^, achieving ≈100% Coulombic efficiency. Notable, the as‐assembled PbO_2_//Sn battery could stabilize cycling for over 1000/115 cycles at 1/10 mAh cm^‒2^, respectively. The future development of PbO_2_//Sn batteries will provide a valuable supplement to the lead‐acid battery industry, reducing lead usage by 50% and supporting more sustainable energy storage.

In recent years, the development of proton battery materials has introduced new possibilities for high‐rate energy storage.^[^ ^86^ ^]^ Hydrated protons have a light mass (19 g mol^‒1^) and small size (≈1.0 Å), and they do not require desolvation, enabling faster ion transport and efficient intercalation/deintercalation kinetics within electrodes.^[^ ^49^, ^82^ ^]^ Additionally, protons can migrate through the Grotthuss mechanism (Figure 8d), hopping along water molecule chains, which minimizes volume expansion during the intercalation process, thus enhancing both rate capability and cycling stability (up to 0.73 million cycles at 4000 C).^[^ ^49b^ ^]^ However, the limitations of the potential and kinetics of existing anodes in acidic electrolytes have hindered the further development of proton batteries. The development of Sn metal addresses this gap, and the relatively low voltage of Sn is well‐suited to existing proton battery cathodes (Figure 8e), enabling the assembly of metal‐proton batteries with reasonable output voltages. Furthermore, the relatively fast stripping/plating kinetics of Sn allow for excellent rate performance in proton//Sn batteries. For instance, when pairing the organic electrode tetra‐chloro‐benzoquinone (TCBQ) cathode with Sn anode, stable voltage output was achieved at a 30 C rate with over 4000 cycles (Figure 8f), demonstrating that the development of proton//Sn batteries may open new opportunities for high‐power battery applications.^[^ ^62a^ ^]^ Similar to Zn, Sn could also be able to match carbon‐based materials with either electric double layer capacitance or pseudo capacitance to form Sn‐ion hybrid supercapacitors to provide extra choice for high rate, long‐term energy storage.^[^ ^87^ ^]^

Sn metal anode has been proved to perform well in acidic flow batteries, which can match various acidic catholyte including VO_2_^+^,^[^ ^57^ ^]^ Fe^3+^,^[^ ^58^ ^]^ Mn^3+^,^[^ ^59^ ^]^ etc. Pump‐free dual‐electrode‐free batteries hold even more possibility due to the avoid of electrolyte flow. MnO_2_/Mn^2+^ chemistry (1.24 V vs SHE) in acid is a good cathode candidate that will proceed stripping/plating process during the battery operation, which allows dual‐electrode‐free MnO_2_//Sn batteries to have good energy densities with an output voltage of 1.4 V (Figure 8g).^[^ ^88^ ^]^ In addition, the dual‐electrode‐free MnO_2_//Sn batteries exhibit good cycling stability due to the stripping/plating mechanism occurring at both electrodes (Figure 8h).^[^ ^18^ ^]^ However, further development and optimization are still required for full potential realization. To further boost the energy density of acidic SnMB systems, the cathode with higher energy density must be applied, such as oxygen in the air.^[^ ^89^ ^]^

### 6.2 Alkaline Sn Metal Batteries

**Figure 9:**
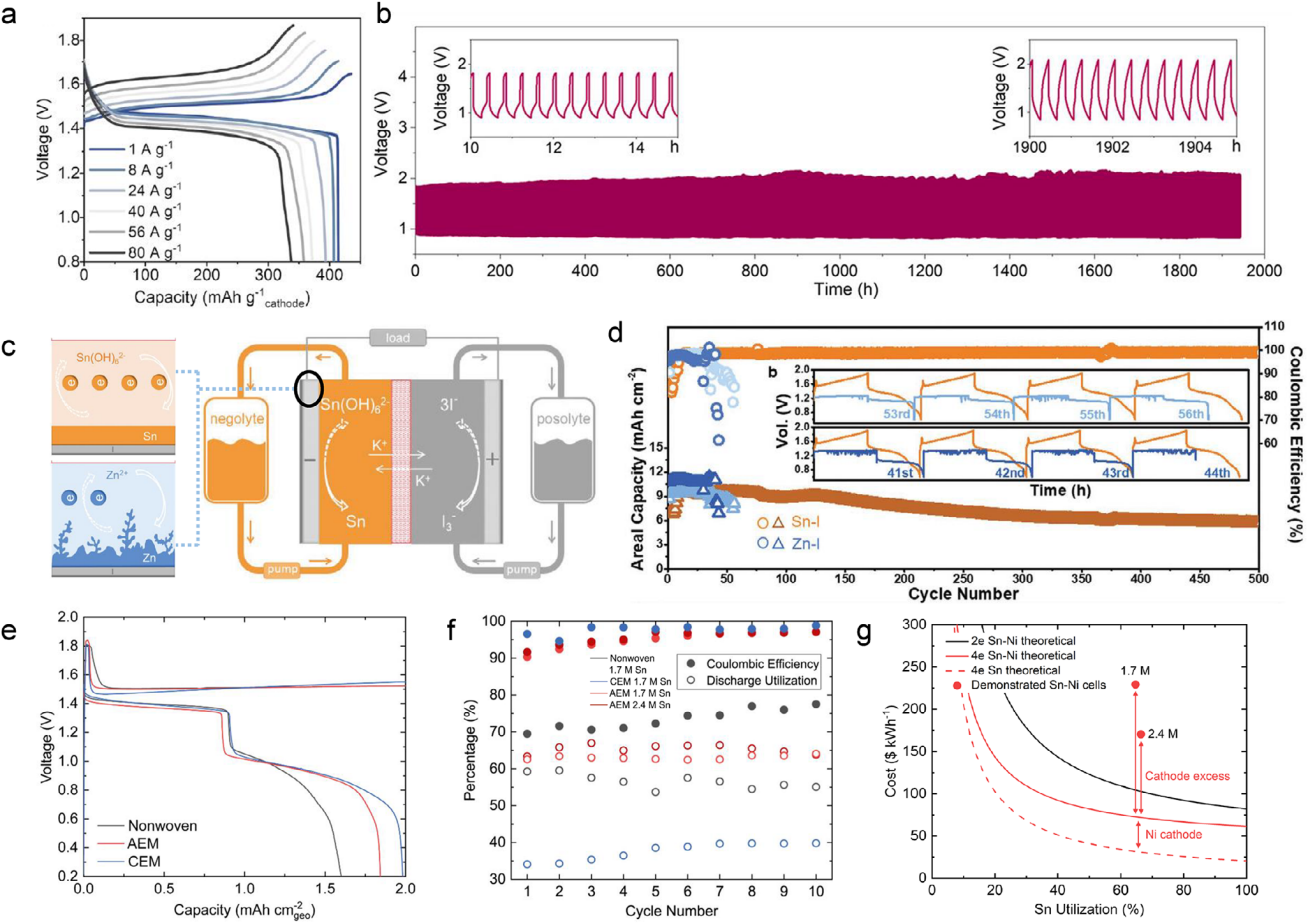
a) Charge/discharge profiles of the Ni(OH)_2_//Sn SnMB at different rates. b) Cycling performance of the Air//Sn SnMB at 5 mA cm^−2^ and 1 mA h cm^−2^. Reproduced with permission.^[^ ^53b^ ^]^ Copyright 2023, American Chemical Society. c) Schematic illustration of comparison between traditional Zn–I redox flow batteries and the Sn–I redox flow batteries based on four‐electron transfer. d) Areal capacity and Coulombic efficiency retention of Sn–I and Zn–I static cells at 5 mA cm^−2^ under 60 °C. Reproduced with permission.^[^ ^20a^ ^]^ Copyright 2021, Wiley‐VCH. e) Voltage profiles of Ni(OH)_2_//Sn pouch cells at 1 mA cm^‒2^ and 2 mA h cm^−2^ with different separators. f) Coulombic efficiencies and Sn utilization retention based on total Sn in the electrolyte for 40 mAh cm^−2^ pouch cell. g) Energy cost as a function of utilization of Sn anode with different chemistries. Reproduced with permission.^[^ ^55^ ^]^ Copyright 2024, Elsevier.

Aqueous alkaline batteries have gained attention due to their inherent safety, low cost, and environmental friendliness.^[^ ^90^ ^]^ The alkaline system holds well‐developed bi‐functional catalysts for oxygen chemistry, which could enable high power air battery chemistries.^[^ ^91^ ^]^ In addition, the alkaline environment is less corrosive to certain electrode materials compared with acid, allowing a wider range of electrode materials (e.g., MnO_2_,^[^ ^92^ ^]^ Ni(OH)_2_,^[^ ^50e^ ^]^ Ni_3_S_2_,^[^ ^93^ ^]^ Co_3_O_4_,^[^ ^50d^ ^]^ AgO,^[^ ^50a^ ^]^ etc.). Alkaline MnO_2_//Zn dry cell is the most commonly used primary battery in our daily life, and researchers are contributing their efforts to make it rechargeable.^[^ ^94^ ^]^ Up to now, various rechargeable aqueous alkaline batteries are well developed based on different metal anodes such as Fe,^[^ ^11^ ^]^ Cd,^[^ ^15^ ^]^ Sb,^[^ ^12^ ^]^ Bi,^[^ ^43^ ^]^ and especially Zn.^[^ ^50^, ^95^ ^]^ Although significant breakthroughs have been made in recent years, and star systems like Ni(OH)_2_//Zn and air//Zn batteries have seen substantial development, these metal anodes are still facing severe critical problems including but not limited to dendrite formation, hydrogen evolution, passivation, and corrosion.^[^ ^16b^ ^]^ Sn metal holds significant advantages compared to other metal anode on its characteristic of dendrite‐less and HER inertness, which usually exhibit better cycling stability over other metal batteries (e.g., Zn). Two‐electron transfer Sn chemistry with extremely low polarization (<25 mV) has already proven to be highly reversible in alkaline systems by multiple published studies.^[^ ^53^ ^]^ When applying Sn metal as an anode in alkaline Ni(OH)_2_‐based batteries, it could deliver nearly 100% DOD (based on the anode) with good capacity retention under different rate conditions from 1 to 80 A g^−1^ (**Figure** **9**a). Benefiting from the low redox potential of Sn(OH)_3_^−^/Sn (−1.0 V vs SHE), the SnMB with Ni(OH)_2_ cathode owns an output voltage of 1.45 V, leading to a promising energy density of 314 W h kg^−1^ based on cathode and anode. After cycling, the tin metal anode showed no noticeable dendrite formation due to its isotropic growth mode, in stark contrast to zinc, which exhibited significant dendrites after just a few cycles. An alkaline air//Sn battery with an output voltage ≈1.0 V could even hold higher energy density while maintaining advance stability (Figure 9b). With the MnO_2_/active carbon air cathode, the air//Sn battery exhibits an energy density of 420 W h kg^−1^ based on the total weight of anodic Sn and the cathodic catalyst or 165 W h kg^−1^ when the weight of electrolyte and current collector into consideration. Not surprisingly, the air//Sn battery remains stable for ≈2000 h at high DOD conditions (99.5%, based on the anode), positioning it as a strong competitor to the zinc‐air battery.^[^ ^53b^ ^]^

The redox potential difference of Sn(IV)‐Sn(0) and Sn(II)‐Sn(0) in alkaline (47 mV higher for four‐electron transfer) is much smaller than that in acid (290 mV for four‐electron transfer), so it will make much more sense to achieving four‐electron transfer Sn chemistry in alkaline batteries. Corresponding Sn(OH)_6_^2−^/Sn reaction could exhibit doubled theoretical anode capacity (903.2 mAh g^−1^ and 6562.5 Ah L^−1^). Moreover, the solubility of Sn(OH)_6_^2−^ is higher than Sn(OH)_3_^−^ (2.8 M vs 0.3 M at 25 °C), which may result in higher energy density. Lu and her co‐workers identified the potential of this four‐electron transfer Sn chemistry and used it as an anolyte for redox flow batteries (Figure 9c).^[^ ^20a^ ^]^ Reversible Sn stripping/plating was activated through a high temperature of 60 °C, then a promising volumetric capacity of 148.61 Ah L^−1^ anolyte (73.07 mAh cm^−2^) was achieved at an average discharge voltage of 1.30 V for 350 h. Compared with the Zn anode in traditional Zn–I redox flow battery suffering from severe dendrite formation, Sn prefers to grow as polyhedron particles isotropically instead of forming a dendrite‐like structure, contributing to higher long‐term stability (Figure 9d). Recently, room‐temperature reversible Sn metal anode with four‐electron redox chemistry was demonstrated in Ni(OH)_2_‐based SnMB by using lean electrolyte and ion exchange membrane to avoid dilute and shuttle of Sn(OH)_3_^−^ redox intermediate.^[^ ^55^ ^]^ Two plateaus at 1.4 and 1.0 V were observed in the discharging voltage profile, corresponding to Sn(OH)_6_^2−^/Sn(OH)_3_^−^ and Sn(OH)_3_^−^/Sn reaction, respectively (Figure 9e). In a SnMB pouch cell with an anion exchange membrane, a Coulombic efficiency of up to 97% was achieved with a high Sn utilization rate (Figure 9f). Furthermore, SnMBs utilizing a four‐electron Sn metal anode demonstrate reasonable energy costs compared to the two‐electron Sn anode (Figure 9g), with an estimated cost of 168 $ kWh^−1^ for Ni(OH)_2_//Sn batteries (the cost of AEM separator is not included). The potential of alkaline SnMBs utilizing a four‐electron Sn anode may be improved by integrating an air cathode in the future, though this will require further research efforts.

### 6.3 Potential of Sn Metal Batteries

**Figure 10:**
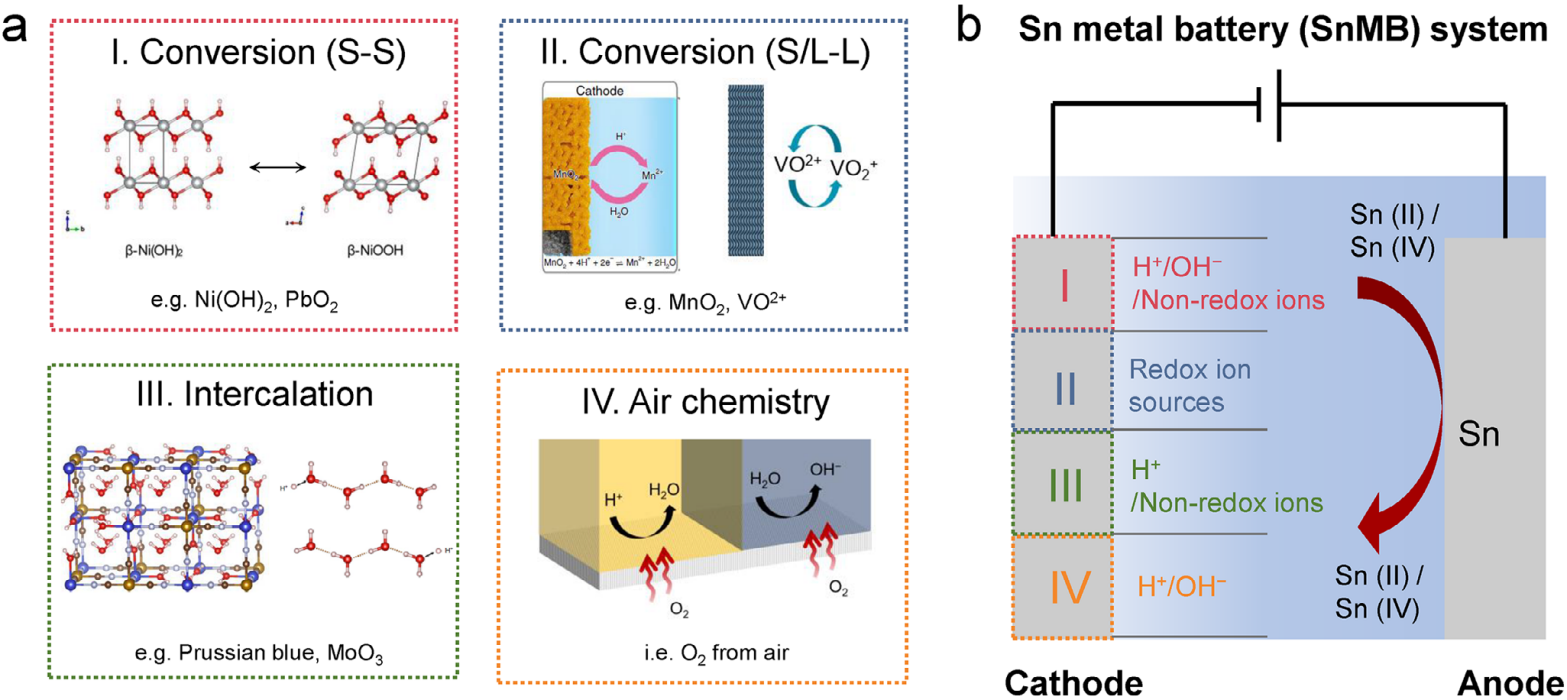
The schematic of the SnMB operation process with different types of cathodes (I to IV). The ion types shown near the cathode in the figure on the left panel correspond to the specific ions required in the electrolyte for each respective cathode type. S means solid phase and L means liquid phase. Reproduced with permission.^[^ ^49^, ^88^, ^96^ ^]^ Copyright 2015, Elsevier. Copyright 2020, Springer Nature. Copyright 2021, Springer Nature.

The current existing SnMBs can be identified as dual ion batteries, or hybrid batteries because Sn‐containing ions so far do not participate in the anodic redox chemistries with the presently available cathode chemistries, although rocking‐chair Sn‐ion batteries may become possible in the future. Batteries with similar mechanisms are common, such as Ni(OH)_2_//Zn batteries, redox flow batteries, proton‐metal batteries, etc. When we evaluate the performance and applicability of different emerging SnMB systems, holistic analysis of all the components (cathode, anode, electrolyte, redox chemistry) is needed. Four anodic reaction options to match the cathodic process of current SnMBs are summarized in **Figure** **10**, including solid‐to‐solid (S‐S) conversion,^[^ ^96^ ^]^ solid/liquid‐to‐liquid (S/L‐L) conversion,^[^ ^88b^ ^]^ ion intercalation and air redox.^[^ ^49b^ ^]^ For aqueous Sn batteries to operate under extreme pH conditions, acid or alkali needs to be added to the electrolyte in addition to the Sn salt (both tin hydroxide and stannic acid have limited solubility in water). The extra amount of acid/alkali or Sn salt in the electrolyte will reduce the energy density at the cell‐level, while an insufficient amount may cause the electrode performance degradation.

For S‐S conversion (e.g., Ni(OH)_2_, PbO_2_) and intercalation (e.g., Prussian blue analogue, MoO_3_) cathodes, proton/hydroxide or non‐redox anions participate in the redox processes. Under such circumstances, the overall energy delivered by the cathode has only a weak correlation to the electrolyte amount. The lean electrolyte is preferred to increase the energy density while proving sufficient io transport, so the electrode/electrolyte ratio needs to be optimized. Although proton intercalation cathode provides an excellent prospect for SnMBs due to its fast kinetics governed by the Grotthuss mechanism, enhancing the specific capacity of the cathode is essential to position such SnMB for future electric mobility applications. The energy density of SnMB with S/L‐L conversion cathode (e.g., MnO_2_, catholyte species in flow batteries) faces more challenges due to active material dissolution in the electrolyte. Researchers may need to pay attention to the utilization rate of at least two kinds of redox species during the performance optimization, and the energy density is significantly relevant to the electrolyte amount and redox active species for both cathode and anode. Fortunately, many S/L‐L cathodes have high specific capacity (> 600 mAh g^−1^) and good solubility, enabling a good scalability of SnMBs for grid energy storage. Taking static acidic MnO_2_//Sn battery as an example, MnSO_4_ maintains a reasonable solubility in water (4.5 M, 241.2 Ah L^‒1^_catholyte_). Ideally, if the volume of the electrolyte is limited to 300 µL cm^‒2^ with the 2 m concentration of both Mn^2+^ (MnSO_4_) and Sn^2+^ (SnMSA_2_), the theoretical energy density based on active materials and electrolyte could reach 149 Wh kg^‒1^. The energy density of SnMBs could even be further boosted with an air cathode to release the mass of cathode active materials.

Metallic Sn anode follows a stripping/plating mechanism during the reversible energy storage and the associated energy density is mainly dependent on Sn salt solubility in the anolyte. For example, SnSO_4_ in sulfuric acid is a commonly used electrolyte solute in industrial electrodeposition of Sn. This system has also been employed in early‐stage studies on Sn metal anode, offering a volumetric capacity of > 30 Ah L^‒1^_anolyte_. New anolytes with higher energies are still under development, such as stannous methylsulfonate in methanesulfonic acid (>2.5 M, 135 Ah L^‒1^_anolyte_). In fact, many tin salts including stannous chloride (>4.4 M, 235.8 Ah L^‒1^_anolyte_), stannous sulfate (>1.5 M, 80.4 Ah L^‒1^_anolyte_), and stannous tetrafluoroborate (>2.2 M, 117.9 Ah L^‒1^_anolyte_) hold higher theoretical solubility in water at room temperature, leading to the possibility of higher energy density via electrolyte composition optimization.

## 7 Conclusion and Perspective

For nearly 15 years research on aqueous Sn metal batteries has been focused on identifying the potential of this system, stabilizing the Sn metal anode, and matching the appropriate cathode to develop full batteries with good performances. Significant advancements have been made in the evolution of Sn‐based systems, transitioning from limited cycle life to stable long‐term cycling, and from early misunderstandings of its electrochemical mechanisms to a more comprehensive understanding of its reaction and deposition behavior with different electron transfer numbers. These advancements have expanded the potential applications of tin, a historically well‐known metal, in energy storage systems. However, significant challenges remain in the development of SnMBs, particularly in regulating the metal growth, optimizing the electrolyte composition, and refining overall cell design.

Although the development of aqueous Sn batteries has progressed rapidly in recent years, they are still in the preliminary stages of research, and many potential bottleneck issues need to be noticed and addressed. The unique growth mode of Sn makes it less prone to forming sharp dendrites, but its polyhedral growth can introduce other challenges that affect battery stability. First, although the isotropic particles are considered less dangerous than sharp dendrites formed by Li and Zn, the edges and corners of large particles can still damage the separator and introduce the risk of short circuits. Second, the increased surface area of isolated particles will also bring a larger electrochemical active surface leading to more serious hydrogen evolution. Additionally, although not widely reported, Sn is able to, under certain conditions, grow rapidly in the [101] direction bounded by faces (101) and (100) to form pyramid needle chains with the potential for short‐circuiting.^[^ ^97^ ^]^ These issues may be less apparent at low areal capacities or low current densities, but at higher capacities, excessive accumulation of dead tin and continuous consumption of active material in the electrolyte can easily lead to battery failure. A more compact, uniform deposit on either planar or non‐planar substrate with controlled thickness is the ideal direction for Sn electrodeposition, which is potentially achieved through metal–substrate bonding, artificial SEI layer, solvation structure optimization, 3D substrate design, etc. In addition, Sn metal deposition under ultrahigh current density (>10 C) or large capacity loading (>10 mAh cm^−2^) requires more study and refinement to better fit the application scenario of fast charging (e.g., proton//Sn battery, Sn‐ion hybrid supercapacitors) and grid‐scale energy storage (e.g., MnO_2_//Sn battery, Sn flow battery, air//Sn battery). Although Sn, as a sp metal, possesses inherent hydrogen evolution resistance, further suppression of hydrogen production is essential to achieve practical‐level Coulombic efficiencies exceeding 99.9%, particularly under extreme operating conditions.

The electrolyte is another critical factor that needs attention in the development of aqueous SnMBs. Interestingly, the saturation concentration of tin salts in the electrolyte, whether acidic or alkaline, is significantly influenced by the counter ions. For example, replacing the sulfate anion in tin salts with methylsulfonate increases the solubility of tin ions by more than four times under the same acidity conditions. While basic research on Sn electrodeposition using stannous sulfate is encouraged due to its status as one of the most established tin electroplating systems and the familiarity with sulfate ions from researchers, which allows more focus on Sn ion behavior, the low solubility of tin sulfate may become a bottleneck for further improving the energy density of SnMBs. Developing new low‐cost, sustainable electrolyte systems with high Sn ion solubility, wide electrochemical stability windows, and negative effect‐free on cathode materials is a key direction for the future development of SnMBs. Additionally, the ease with which divalent tin ions are oxidized by oxygen in the air makes the oxidation resistance of stannous electrolytes crucial to preventing self‐degradation. Inspired by the tin electroplating industry, adding antioxidants like hydroquinone and methylsulfonate to the electrolyte is a potential direction.^[^ ^98^ ^]^

Among the emerging static Sn battery systems, air//Sn batteries, proton//Sn batteries, manganese dioxide//Sn batteries, lead dioxide//Sn batteries, and nickel hydroxide//Sn batteries are representative systems that show significant potential in one or more battery performance aspects. When modifying the anode, electrolyte, or separator, it is essential to tailor these modifications to the appropriate full‐cell systems and thoroughly investigate their effects on all battery components. The development of corresponding cathodes, such as research into bifunctional oxygen evolution/oxygen reduction catalysts, the development of high‐capacity proton battery cathodes, and large areal capacity manganese dioxide cathodes, will play a crucial role in advancing Sn battery technology. In addition, Sn‐based flow batteries have the potential to emerge as strong competitors to traditional metal‐based flow batteries, such as Zn‐Br_2_ and Zn‐I_2_ systems.^[^ ^99^ ^]^ Halogen catholytes are promising to match with Sn anodes in both acidic and alkaline conditions. The ion exchange membrane can be adjusted to meet the pH requirements of the catholyte without the need for a bipolar membrane.

Aqueous Sn metal battery is a technology full of both challenges and promises. The unique properties of tin offer significant potential for long‐term, sustainable energy storage for both grid and daily use. Compared to other widely studied aqueous metal anodes such as zinc, tin exhibits less tendency of dendrite formation and a higher hydrogen evolution overpotential, positioning it as a promising candidate for long‐lifespan aqueous battery systems. For researchers, what matters more than the market price is the actual reserve and the potential price considering the resources, mining, and processing, and difficulty/easiness in extraction. Although the cost of tin still remains higher than zinc, this cost disparity may be mitigated as Sn batteries achieve extended lifetimes, potentially lowering the per‐cycle energy storage cost relative to zinc batteries. Furthermore, the recycling ratio of Sn is less than 35% of total usage, leaving significant room for improvement. Advancements in recycling technology could further optimize the process and reduce recycling costs. Additionally, if industries adopt cheaper alternatives such as aluminum or plastics to replace Sn in applications like packaging and release the usage of Sn, the energy cost of SnMBs may further decrease. With a stable global supply chain and relatively low price volatility, Sn is well‐positioned to contribute significantly to the 100 TWh energy storage systems required for a sustainable future.^[^ ^100^ ^]^ We hope that continued research into this field will unlock the full potential of aqueous Sn metal batteries, answering key questions and paving the way for their future commercial adoption.

## Conflict of Interest

The authors declare no conflict of interest.
